# 13^th^ European Headache Federation Congress 2019 - Abstracts: May 30^th^ to June 1^st^ 2019. Athens, Greece

**DOI:** 10.1186/s10194-019-1049-1

**Published:** 2019-12-10

## A01 Rationale and design for a randomised, single-centre, double-blind, sham-controlled study of non-invasive vagus nerve stimulation for the acute and preventive treatment of post-traumatic headache

### Bert Vargas^1^, Eric Liebler^2^, Stephen Bunt^1^, Charlene Supnet^1^

#### ^1^University of Texas Southwestern Medical Center, Dallas, Texas; ^2^electroCore, Inc., Basking Ridge, New Jersey

##### **Correspondence:** Bert Vargas(bert.vargas@UTSouthwestern.edu); Eric Liebler(eric.liebler@electrocore.com)

**Background:** Worldwide, ~69 million people per year sustain a traumatic brain injury (TBI); many develop post-traumatic headache (PTH). There has been little study of treatments for mild TBI (mTBI) or PTH, and clinicians often use drugs approved for primary headache disorders. Many patients self-treat with OTC agents or NSAIDs but have

suboptimal pain relief. We initiated a randomised, double-blind (DB), sham-controlled, parallel-group pilot study of non-invasive vagus nerve stimulation (nVNS) for acute and preventive treatment of PTH.

**Methods:** The study is enrolling adults who present 1-4 wks after a head injury, meet ICHD-3 criteria for acute headache attributed to mTBI, and have ≥2 headaches/wk with a migraine or probable migraine phenotype. After a 2-wk run-in period, subjects will be randomly assigned (1:1 allocation) to receive daily preventive therapy and as-needed acute treatment with nVNS or a sham device. Preventive therapy will consist of two 120-second stimulations given 3 times daily. Acute treatment will comprise 2 stimulations administered at headache onset, followed by 2 stimulations given 20 min after the start of initial treatment. Subjects are not to use acute rescue medication for 120 min post-treatment. The primary effectiveness endpoint is the decrease in pain (on a 7-point numeric scale) at 60 min post-treatment for all treated headache attacks. Secondary endpoints include decrease in the frequency of headache days between the run-in period and the last 2 wks of the DB period and responder rates (ie, percentages of subjects with ≥50% decrease in attack frequency). The primary safety endpoint is the incidence of treatment-related serious adverse events.

**Results:** Up to 80 subjects will be enrolled at 1 North American site. The expected duration is 12 mos (9 mos for enrolment, 14 wks for active participation).

**Discussion:** This study is designed to assess the efficacy and safety of nVNS as a novel acute and preventive therapy for PTH.

**Author Disclosures**

**B. Vargas** has received advisory board fees from Amgen, Novartis, Allergan, Alder, Teva, Lilly, Upsher-Smith, Biohaven, Promius, and Xoc and has received speaker fees from ATI. He serves on the board of directors for the American Headache Society and the Headache Cooperative of the Pacific and is an editorial board member for *Neurology Today*.

**E. Liebler** is an employee of electroCore, Inc., and receives stock ownership.

**S. Bunt** has no financial conflicts of interest to declare.

**C. Supnet** has no financial conflicts of interest to declare.

## A02 No increase in brain iron content in refractory chronic migraine: a quantitative susceptibility mapping study

### Mi Ji Lee^1,*^, Seulki Yoo^2,3,*^, Soonwook Kwon^1^, Soohyun Cho^1^, Seung-Kyun Lee^2,3^, Chin-Sang Chung^1^

#### ^1^Department of Neurology, Samsung Medical Center, Sungkyunkwan University School of Medicine, Seoul 06351, South Korea; ^2^Department of Biomedical Engineering, Sungkyunkwan University, Suwon 16419, South Korea; ^3^Center for Neuroscience Imaging Research, Institute for Basic Science (IBS), Suwon 16419, South Korea

##### **Correspondence:** Mi Ji Lee(mirony.lee@gmail.com)

*These two authors contributed equally.

**Abstract**

**Background:** Iron accumulation in the periaqueductal gray and basal ganglia has been reported in patients with migraine. However, results from previous studies were challenged due to methodological issues. We performed quantitative susceptibility mapping (QSM), a state-of-art MRI technique, to measure iron content in patients with refractory chronic migraine.

**Methods**: We recruited patients with refractory chronic migraine and age-sex-matched headache-free controls. Using a 3T MR scanner, QSM were obtained from a conventional multi-echo gradient-echo sequence. Caudate nucleus, globus pallidus externa (GPe), globus pallidus interna (GPi), red nucleus, substantia nigra, and periaqueductal gray were manually segmented for each participant. Susceptibility values and R2* values of segmented areas were calculated and compared between groups. Wilcoxon signed-rank test was applied to test the paired group difference.

**Results:** A total of 10 patients with refractory chronic migraine and 10 age-sex-matched headache-free controls were recruited. Total susceptibility values were not different between groups in caudate nucleus, GPe, GPi, red nucleus, substantia nigra, and periaqueductal gray. Mean susceptibility values were not different in all segmented regions but GPe, where patients showed decreased values than controls (p=0.009). R2* values were also not different between groups.

**Conclusion:** Using QSM, we found that brain iron content is not increased in patients with refractory chronic migraine. Pathophysiology of chronic migraine may not be associated with iron metabolism or irreversible damage to specific nuclei.

## A03 Typical migraine aura without headache in Korean headache clinic

### Byung-Kun Kim

#### Department of Neurology, Eulji Hospital, Seoul, Korea

##### **Correspondence:** Byung-Kun Kim(kbk1403@eulji.ac.kr)

**Background:** Typical aura without headache is a type of migraine with typical aura, which has been rarely reported. The reported prevalence of typical aura without headache is different one from another ranging 0.2-6.5%. The prevalence and characteristics of Korean patients with typical aura without headache are not known. The aim of this study was to investigate the prevalence and characteristics of typical aura without headache in Korean Headache Clinic.

**Methods:** The types of aura and headache followed by aura were analyzed in 214 patients with typical aura at the Headache Clinic of Eulji Hospital from 2010 to 2018. We diagnosed migraine with typical aura according to the diagnostic criteria of International Classification of Headache Disorders.

**Results:** All the patients have visual aura. Twenty-four (11.2%) patients have other types of aura (sensory aura in 8.4% and aphasic aura in 7.5%). Typical aura without headache was observed in 15.0% (32 patients, with a male:female of 1:1.7 and a median age of 35 years) of migraine with typical aura. Among them, 25% (8/32) have typical aura without headache only (pure form) and other patients have both typical aura without headache and typical aura with headache. Typical aura without headache group was older than typical aura with headache group (median age of 28 years)

**Discussion:** These data suggest that pure typical aura without headache is a rare migraine subtype in headache clinics in Korea.

## A04 Factors reducing depressive symptoms in depressed migraine patients

### Sung-Pa Park

#### Department of Neurology, School of Medicine, Kyungpook National University, Daegu, Korea

##### **Correspondence:** Sung-Pa Park(sppark@mail.knu.ac.kr)

**Background**

About 40 to 50% of migraine patients have a comorbid depression. However, there is no evidence-based medicine for treating depressive symptoms in depressed migraine patients. We identified factors reducing depressive symptoms in these patients.

**Methods**

We reviewed electronic data which collected new patients with migraine in our headache clinic. A total of 604 patients were found. Among them, we included patients who had depression at an initial visit (T1) and who could be followed at 12 weeks (T2). All patients conducted the Patient Health Questionnaire-9 (PHQ-9) at two periods. We stipulated that 8 or more of the PHQ-9 score suggest depression. We examined factors to be associated with the decrement of the PHQ-9 score during 12 weeks.

**Results**

A total of 502 patients were excluded in the review due to having no depression (n=398) at T1 and no visit at T2 (n=104). Finally, 102 depressed patients were included in the study. Of them, 24 patients (23.5%) had medication overuse headache (MOH) and 36 (35.3%) received psychiatric drugs including antidepressants and anxiolytics. All took preventive drugs at T2. Mean score of the PHQ-9 at T1 was significantly decreased at T2 (*p* < 0.001). Mean difference of the PHQ-9 score was correlated with MOH and mean differences of attack frequency, headache day, and the VAS score by univariate analyses. However, the type of preventive drugs and the intake of psychiatric drugs were not associated. The strongest factor by multivariate analyses was mean difference of headache day (*ß* = 0.345, *p* < 0.001) followed by MOH (*ß* = 0.249, *p* = 0.008).

**Discussion**

The improvement of depressive symptoms in depressed migraine patients may rely on the headache management instead of the use of psychiatric drugs.

## A05 AHS Abstract: The association between occurrence of migraine headache and objectively-assessed sleep among adults with episodic migraine: A Prospective Cohort Study

### Angeliki Vgontzas^1,3^ , Wenyuan Li^2,4^, Elizabeth Mostofsky^4^, Murray A. Mittleman^2,3,4^, Suzanne Bertisch^1,2,3^,

#### ^1^Brigham and Women’s Hospital, Boston, M;^2^Beth Israel Deaconess Medical Center, Boston, MA; ^3^Harvard Medical School, Boston, MA; ^4^Harvard T.H. Chan School of Public Health, Boston, MA

##### **Correspondence:** Angeliki Vgontzas(avgontzas@bwh.harvard.edu)

**Background:** Patients with migraine frequently report sleep disturbance, including difficulty falling sleep and shorter sleep duration. There are sparse data examining the temporal association between daily headaches and subsequent sleep. Given the burden of sleep disturbance in patients with migraine, we examined the association between migraine headache and subsequent sleep duration and fragmentation.

**Methods:** We conducted a prospective cohort study of 98 adults with episodic migraine. Participants reported headaches, sleep, and health habits on daily electronic diaries and wore actigraphs for 6 weeks. Migraine was defined by ICHD-3 criteria and diagnoses were reviewed by clinicians. Sleep measurements included total sleep time, sleep efficiency (proportion of sleep/rest period) and wake after sleep onset (minutes awake from sleep onset until wake time, WASO). We examined whether days with migraine headache were associated with sleep that night, using adjusted multivariable linear mixed models.

**Results:** Participants were a mean age of 35.1±12.1 years, 87.7% female, and averaged 5 headaches/month. Over 4406 days, we observed 1077 headache days, with an average duration of 8.8 hours. Over the course of the study, nightly objective sleep duration was 7.3 ±1.4 hours, sleep efficiency was 89.6±4.6% and WASO was 44.7±24.1 minutes. Objective sleep duration was 7.3 minutes (95% CI:1.5, 13.0) longer on nights following a migraine headache day compared to nights following a headache-free day. Sleep efficiency and WASO were not significantly different on nights following migraine headache days compared to nights following headache-free days (sleep efficiency: -0.06 min, 95% CI: -0.3, 0.2; WASO 1.5 min, 95% CI: 0.0, 3.0).

**Conclusions**: Headache attacks are unlikely to explain the sleep disruption in patients with episodic migraine, which suggests reported sleep disturbance may represent an endophenotype of migraine.

## A06 Sleep characteristics and pain sensitivity in episodic and chronic migraine and tension-type headache (TTH) - a population study

### Angeliki Vgontzas^1,3^, Suzanne Bertisch^1,2,3^, Monika Haack^2,3^, Rigmor Jensen^4^, Lars Bendtsen^4^, Richard B. Lipton^5^, Sait Ashina^2,3^

#### ^1^Brigham and Women’s Hospital, Boston, MA; USA; ^2^Beth Israel Deaconess Medical Center, Boston, MA, USA; ^3^Harvard Medical School, Boston, MA; USA; ^4^Danish Headache Center and Department of Neurology, University of Copenhagen, Glostrup Hospital, Denmark; ^5^Department of Neurology, Montefiore Headache Center and Department of Epidemiology and Population Health, Albert Einstein College of Medicine, NY, USA

##### **Correspondence:** Angeliki Vgontzas(avgontzas@bwh.harvard.edu)

**Background:** Sleep disturbances are commonly reported by those with primary headache disorders. Short or disturbed sleep is associated with lowering of pain thresholds, although the majority of studies have been limited to individuals without headache. Our aim was to assess whether self-reported sleep characteristics in those with migraine and TTH in a population-based sample were associated with increased pain sensitivity.

**Methods:** A sample of 644 adult individuals underwent a headache interview (using ICHD criteria) and completed a questionnaire that included four items on sleep (self-reported duration, sleeping problem, snoring and feeling well-rested). A total of 314 participants also underwent concurrent pain testing, including a pericranial total tenderness score (TTS), and cephalic and extracephalic pressure pain thresholds (PPTs). Data were analyzed using adjusted logistic or linear regression.

**Results:** The sample was comprised of 40.9% headache sufferers (22.8% migraine, 17.1% TTH), of whom 12.5% had chronic headache (CH). Compared to non-headache sufferers, those with headache were more likely to report a sleep problem [OR=2.8(1.7-4.5)] and not feeling well-rested [OR=3.0(2.1-4.4)]. There was no difference in average sleep duration or snoring. TTS, but not PPTs, was higher in those with sleep problems and this difference was greater for CH (26.7, p=0.014) than for EH (19.1, p=0.051) compared to controls (14.0, ref). Similar results were found for those not well rested, with a higher TTS in those with CH (25.3, p<0.001) and EH (19.5, p=0.015) compared to controls (12.0, ref).

**Conclusion:** Migraine and TTH are associated with higher rates of self-reported sleep problems and feeling unrested. TTS indicates increased pain sensitivity among those who report more sleep problems and this difference is greater for those with CH. Causal sequence cannot be determined in cross-sectional data. Future work is needed to determine if disturbed sleep modifies pericranial tenderness contributing to overall headache burden.

## A07 Pain Extent is not Related to Widespread Pressure Pain Sensitivity in Men with Episodic Cluster Headache

### García-Azorín D^**1**^, Fuensalida-Novo S^**2**^, Palacios-Ceña M^**2**^, Falla D^**3**^, Gómez-Mayordomo V^**4**^, Cuadrado ML^**4**^, Guerrero AL^**1**^, Fernández-de-las-Peñas C^**2**^, Barbero M^**5**^

#### ^1^Headache Unit. Hospital Clínico Universitario de Valladolid. SPAIN; ^2^Departamento de Fisioterapia, Terapia Ocupacional, Rehabilitación y Medicina Física. Universidad Rey Juan Carlos, Alcorcón, Madrid, SPAIN; ^3^School of Sport, Exercise and Rehabilitation Sciences, College of Life and Environmental Sciences, University of Birmingham, Birmingham, UK; ^4^Neurology Department. Universidad Complutense de Madrid, SPAIN; ^5^Rehabilitation Research Laboratory 2rLab, Department of Business Economics, Health and Social Care, University of Applied Sciences and Arts of Southern Switzerland, Manno, Switzerland

##### **Correspondence:** García-Azorín(davilink@hotmail.com)

**Background**: It is commonly accepted that larger pain extent reflects a clinical sign of central sensitization and enlarged areas of pain have been associated with more severe pain and greater pressure pain sensitivity in some musculoskeletal pain conditions, e.g., knee osteoarthritis or whiplash associated neck pain. However, this association has not been observed in primary headaches such as tension-type headache or migraine. The aim of this study was to investigate whether the perceived pain extent, as assessed from self-reported pain drawings, relates to widespread pressure pain sensitivity, clinical pain and psychological outcomes in men with cluster headache. **Methods**: Forty men with episodic cluster headache reported their pain on four different body charts representing the head/neck which were subsequently digitized allowing pain extent to be calculated utilising a novel software. Pressure pain thresholds (PPT) were assessed bilaterally over the temporalis muscle (trigeminal area), C5-C6 joint (extra-trigeminal area) and tibialis anterior muscle (distant pain-free area). Clinical features (cluster periods/year; number of attacks/day, pain intensity) of the headache, and anxiety/depressive levels (Hospital Anxiety-Depression Scale, HADS) were also assessed. Pearson correlation coefficients were computed to reveal correlations between pain extent and the remaining outcomes. **Results**: No significant associations between pain extent and PPTs in trigeminal, extra-trigeminal and distant pain-free areas, headache features, and psychological variables, e.g., anxiety/depressive levels were found. **Conclusions**: Pain extent in the trigemino-cervical area was not associated with any of the clinical outcomes and not related to the degree of pressure pain sensitization in men with episodic cluster headache.

## A08 Central sensitization in Men with Episodic Cluster Headache

### García-Azorín D^**1**^, Palacios-Ceña M^**2**^, Gómez-Mayordomo V^**3**^, Fuensalida-Novo S^**2**^, González-García N^**3**^, Guerrero AL^**1**^, Cuadrado ML^**3**^, Fernández-de-las-Peñas C^**2**^

#### ^**1**^Headache Unit. Hospital Clínico Universitario de Valladolid. SPAIN; ^2^Departamento de Fisioterapia, Terapia Ocupacional, Rehabilitación y Medicina Física. Universidad Rey Juan Carlos, Alcorcón, Madrid, SPAIN; ^3^Neurology Department. Universidad Complutense de Madrid, SPAIN

##### **Correspondence:** García-Azorín D(davilink@hotmail.com)

**Background:** It is claimed that central sensitization can also play, in conjunction with vascular processes, a relevant role in the pathogenesis of cluster headache. Widespread pressure pain hypersensitivity is a clinical manifestation of sensitization of central pain pathways which has been observed in other primary headaches such as migraine or tension type headache. The aim of this study was to investigate the presence of pressure pain sensitivity within trigeminal (symptomatic) and extra-trigeminal (pain-free) areas in a sample of men with episodic cluster headache. **Methods**: Forty men with episodic cluster headache and 40 matched controls participated. Pressure pain thresholds (PPT) were bilaterally assessed over one trigeminal point (i.e., temporalis muscle), one extra-trigeminal points (i.e., C5/C6 joint), and two distant points (i.e., second metacarpal and tibialis anterior muscle) by an assessor blinded to the subject’s condition. Patients were assessed in a remission phase, at least 1 month from the last cluster attack and without taking preventive medication. **Results**: The analysis found that PPTs were significantly decreased bilaterally over the trigeminal (mean difference: 85-93 kPa), extra-trigeminal (62-79 kPa), and distant (second metacarpal: 72-92 kPa; tibialis anterior: 135-155 kPa) points in men with cluster headache when compared to healthy controls (all, P<0.001). No significant association between widespread PPTs and clinical headache features was found. **Conclusions**: Our findings revealed bilateral widespread sensitivity to pressure pain in men with episodic cluster headache during a remission phase. These results support the presence of sensitization of central pathways in episodic cluster headache although individuals were asymptomatic (remission phase) and without medication.

## A09 Observational, open-label, non-randomized study on the efficacy of onabotulinumtoxinA in the treatment of Nummular Headache: Series of 38 patients:

### García-Azorín D, Sierra A, Trigo J, Martínez-Pías E, Guerrero Al

#### ^1^Headache Unit, Neurology Department. Hospital Clínico Universitario, Valladolid, SPAIN

##### **Correspondence:** García-Azorín D(davilink@hotmail.com)

**BACKGROUND** Nummular headache (NH) is a primary headache representing around 4% of patients in hospital-based series^1^. Neuromodulators or antidepressants are the most used preventives^2^. Small fiber impairment has been described^2^, which represents a potential target for onabotulinumtoxinA (OnabotA)^3^. We aimed to evaluate efficacy of OnabotA in the treatment of NH.

**METHODS** Observational, open-label, non-randomized, study. We included patients older than 18 with IHCD diagnosis of NH, with at least 10 headache days per month. The study was done in a third level lospital from February 2014 to February 2019. We injected 25 units of onabotA. We present data as mean (standard deviation) or median and interquartile range [IQR].

**RESULTS** We included 38 patients with median age of 61.5 [IQR 43.5-75.2] and 63.2% female. Preventive had been used in 26 patients (68.4%). At baseline, the mean number of headache days per month was 23.7 (8.1). Percentage of patients with a 50% response was 63.2% and 81.6% at 12 or 24 weeks. Mean number of headache days per month decreased to 10.6 and 7.0 days at 12 or 24 weeks (p<0.001). Any moderate adverse events occurred.

**DISCUSSION** OnabotA was effective reducing headache days in NH patients. Randomized controlled trials are needed to ascertain the benefit of onabotA in this condition.

**REFERENCES1.** Guerrero-Peral AL et al. Nummular headache with and without exacerbations: Comparative characteristics in a series of 72 patients. Cephalalgia 2012;32(8):649-653.**2.** Cuadrado ML et al. Nummular headache: an update and future prospects. Expert Rev Neurother 2018;18(1):9-19. **3.** Burstein R. Selective inhibition of meningeal nociceptors by botulinum neurotoxin type A: therapeutic implications for migraine and other pains. Cephalalgia 2014;34(11): 853-69.

## A10 A particular case of facial paraesthesias due to a meningioma

### Angelo Torrente^1^, Concetta Rubino^2^

#### ^1^Department of Biomedicine, Neurosciences and Diagnostic (Bi.N.D.), University of Palermo, Italy; ^2^Outpatient Neurological Clinic, P.T.A. “Biondo” A.S.P. Palermo, Italy

##### **Correspondence:** Angelo Torrente(angelotorrente92@gmail.com)

**Objective**: here we aim to show a case of facial paraesthesias due to the uncommon cause of a meningioma.

**Case study**: a 63-years-old-woman came to our outpatient neurological clinic for the gradual onset of paraesthesias to the left half of the superior lip in three months. Her complaint started as a tingling sensation and became progressively more and more intense until turning into a burning and swelling sensation. The sensations were constant, worsened by speaking and reduced during the night. We first prescribed a medical therapy (pregabalin and a supplement drug containing vitamins B1, B6, E and α-lipoic acid). After, the patient underwent a massive facial MR and, later, a brain contrast enhanced (CE) MR. Finally, she underwent a neurosurgical consult, a radiotherapic one and cyberknife intervention.

**Results**: neurological examination was irrelevant, symptoms decreased just a little after medical therapy. The basic massive facial MR showed only an intrasellar arachnoidocele. The following brain CEMR showed an extracranial vascularised formation at the level of left cerebellopontine angle (coronal diameter 13x12mm, axial one 16.7x16.4mm), with marked dural contrast enhancement and associated to compression of the origin of left trigeminal nerve and Gasser’s ganglion. Neurosurgeon suggested to consult a radiotherapist, who set two options: watchful waiting or performing cyberknife intervention; our patient chose the latter option (25Gy in 5 fractions) with a reduction of symptoms (no more swelling sensation and no speech worsening), but persistence of paraesthesias.

**Discussion**: cerebellopontine angle tumours may cause trigeminal neuralgia, but such painful condition is usually brief, intense and electric shock-like. In our case, the meningioma is probably the cause of such particular clinical presentation and cyberknife intervention may have reduced tumour dimensions, soothing symptoms. Since patient did not recovered fully, other approaches are still needed.

Note: authors of this abstract have nothing to disclose.

Written, informed consent for publication was obtained from the patient.

## A11 Digital therapeutics neuromodulation for migraine

### Oved Daniel^1^ Eran chenker^2,3^

#### ^1^Headache & Facial Pain Clinic Director. Ramat Aviv Medical Center. Tel Aviv, Israel; ^2^Management of Health Informatics and Analytics program. Milken Institute School of Public Health. George Washington University. Washington, USA; ^3^Neurolief, Netanya, Israel

##### **Correspondence:** Oved Daniel(danieloved@gmail.com)

**INTRODUCTION**

Relivion® is the first approved CE non-invasive multi-channel brain neuromodulation system for treating neurological and neuropsychiatric disorders. It offers precise, personalized care by delivering stimulation to six branches of the occipital and trigeminal nerves via three adaptive output channels.

**METHODS**

The latest clinical performance and safety of self-administered treatment for migraine using combined occipital and trigeminal neuromodulation utilized the Relivion e-Relief™ mobile App gather treatment data from the device and upload it to a secure cloud database for analysis and treatment optimization. Using its three adaptive channels the Relivion® can deliver various combinations of treatment architectures, among which the best setup for each patient is chosen while the self-learning system continues to adapt and improve the treatment as time goes by.

**RESULTS**

The clinical prospective, randomized, double-blind, parallel-group, sham-controlled, evaluated the clinical performance and safety of the Relivion® in the abortive treatment of episodic and chronic migraine. 55 subjects were randomized. At one-hour post-treatment, the treated group showed an average of 53.1% reduction in pain VAS score compared with only 10.3% in the sham group (P=0.0002). Responders rate was significantly higher in the treatment group compared to sham at 1, 2 and 24 hours post treatment (P<0.05). In the treatment group, 43% of the subjects with severe or moderate baseline pain level were pain-free at two hours post treated compared to only 10.5% in the sham group (P=0.027). At 2-hours, 76.2% of the subjects in the treated group reached Headache Relief compared to 31.6% in the sham group (P= 0.01). No serious adverse event was reported.

**CONCLUSIONS**

The results of the reviewed clinical trials demonstrate that self-administered abortive treatment of migraine by the Relivion® was safe and highly effective.

## A12 Cognitive functions in migraineurs with and without aura

### Konstantinos Kampouris^1^, Dimitrios Kasselimis^2^, Dimos D. Mitsikostas^3^, Constantin Potagas^3^

#### ^1^MSc Clinical Neuropsychologist, Neuropsychology and Rehabilitation Unit for Brain Injury, ELEPAP, Ioannina, Greece; ^2^Neuropsychology and Language Disorders Unit, 1^st^ Neurology Department, Aiginiteion Hospital, National & Kapodistrian University of Athens, Greece; ^3^Associate Professor of Neurology, 1^st^ Neurology Department, Aiginiteion Hospital, National & Kapodistrian University of Athens, Greece

##### **Correspondence:** Konstantinos Kampouris(konstantinos_kab@hotmail.com)

**ABSTRACT**

Aim: To assess cognitive functions in migraine patients (with and without aura) in comparison to a healthy control group. Method: The research sample is divided in two groups: the research group, which consists of 31 migraine patients (10 of them having migraine aura) that fulfill the diagnostic criteria of the ICHD-3 beta version, and the control group, which consists of 30 healthy subjects, matched by age and education years. In this research design, neuropsychological tests are used for the assessment of cognitive functions, while scales and questionnaires are used for the collection of demographic and clinical data. Results: Statistical analyses show that the performance of migraineurs and especially of those without aura in interictal stages is significant lower than the group of healthy subjects in the tasks of Immediate, Delayed Recall and Memory Recognition of the Rey Complex Figure Test (RCFT) and MoCA. In contrast, they score higher than healthy controls in the Beck Depression Inventory (BDI). However, after controlling for the effect of MoCA on the results no main effect of migraine aura is found. Conclusions: In interictal stages migraineurs and especially migraineurs without aura exhibit significant lower performance scores compared to a group of healthy controls in neuropsychological tests of visual memory recall and recognition. However this relationship is fully mediated by the performance scores in MoCA, which indicates a lower status of general cognitive function in that group. More studies are needed, in order to further examine those findings and their association with the mechanisms of migraine.

## A13 Application of micro-cauterisation (on cervical,occipital and temporal areas) for patients with chronic migraine & headache due to medicaltion overuse (MOH). Report of 21 cases

### Papageorgiou E^1^, Kovas K^2^

#### ^1^Chief at the Neurology Department of "G.Gennimatas"General Hospital of Athens; ^2^Resident at the Neurology Department of "G.Gennimatas" General Hospital of Athens

##### **Correspondence:** Kovas K(kovaskonstantinos@gmail.com)

**Backround:** Migraine is a highly disabling disease, usually accompanied by medication overuse with high financial and social impact. Neuromodulation may play an important role in the application of new alternative treatment with microcautery stimulation on sites of cutaneous allodynia for these patients. The present study is based on the theory of the distorted communication within trigeminocervical complex.

**Objectives:** The aim of this study was to evaluate the efficacy of micro-cautery stimulation on patients with chronic migraine -with and without aura-and MOH

Materials and methods: A total of 21 patients with migraine were selected. All of them were chronic migraneurs with poor response to medication. The cauterisation on cervical, occipital and temporal areas, depending on what the patients referred to as the most painful points during the attack of migraine and also during the inter-ictal period, once a week for about 4 months.

**Results:** An evaluation scale was completed at the end of the study for each individual. The majority reported a significant amelioration, in frequency, intensity, duration in pain and a decrease in the use of medication. To be noted that several patients continued the sessions after the end of the study.

**Conclusion:** It seems that the thermal micro-cauterisation may be very useful to assist patients with migraine. Possible mechanisms underlying the effectiveness of the method are discussed.

## A14 USE OF A DIGITAL PLATFORM IN HEADACHE TRAINING. STRENGTHS AND WEAKNESSES IN 5 YEARS

### Luca Maria Messina, Vincenzo Raieli

#### Child Neuropsychiatry Unit - ISMEP - ARNAS Civico Palermo, Italy

##### **Correspondence:** Luca Maria Messina(LMM85@LIBERO.IT)

**OBJECTIVE:** Primary headaches, especially migraine, are disorders with an important impact on public health. Migraine is at the seventh place among the more disabling disorders. It is very important for specialists to have an adequate training for the management of headaches. The increasing development of social networks and in general of digital instruments led us to implement our project of a digital platform only focused on headaches, started in 2014 with the technological support of an industrial sponsor (Janssen). We monitored the activity of the platform in the following years, publishing some works (1-3) that emphasized its strengths and weaknesses. The aim of the current study is to analyze strengths and limits of a digital platform in headache training after 5 years.

**METHODS:** Our platform is totally free and easily accessible to doctors after registration and approval of the group’s administrators. It is possible to access using website or smartphone app. It is divided in a section for pediatric and a section for adult headaches and contains more than 270 resources totally concerning headache. Each member can download or upload contents and discuss clinical cases. Users can choose to receive notifications for each new contribution via email. We have also created a WhatsApp group to alert members about new contributions and to encourage real-time communication. Administrators can follow the members’ activity (number of access, trend topics, uploaded/downloaded resources, discussions, average download for user). We monitored the evolution of the platform’s activities over time and compared our experience with other digital platforms.

**RESULTS:** To this day the section dedicated to adult headaches includes 60 members and each did 14 downloads on average. The section dedicated to pediatric headaches includes 67 members with average of 19 downloads. We highlighted a progressive increase in the number of users since the start of our project and an increase in the number of downloads simultaneously with upload of new resources. The items that most capture the attention are PowerPoint presentations and resources closely related to common clinical cases or with short reading time. Below the top ten of the downloaded articles:

**Table:** 

Resource	Downloads
Headache treatment PowerPoint	72
Article: When to investigate headache	69
Master in headache PowerPoint	48
Migraine aura videos	37
Innovation in migraine	36
Continuum neurology	36
Dysphagia and neuromuscular disease PowerPoint	31
Secondary headaches PowerPoint	29
Group IV headaches (according to ICHD III) PwP	23
Secondary headaches algorithm	22

The data collected during this monitoring period also showed that the number of uploads remains low and appears considerable the difference with the number of downloads. Most of the resources were uploaded mainly by the administrator (about 90%) and by a few other users (5 users). In general there are few discussed cases and the activity of the platform members appears to be considerably reduced during the summer months. Even the Whatsapp group has not significantly increased the number of active members. Finally, there is not a great difference in activity between specialists enrolled in a scientific society dedicated to headaches and those enrolled in other societies (neurologists, childhood neuropsychiatrists).

Some of these data are quickly summarized in the following chart, in which the blue dot indicates the total number of downloads and the red dot indicates the total number of uploads in that month:

**CONCLUSIONS**: The increase in subscriptions shows that our social network dedicated to headaches is appreciated by the scientific community. Nevertheless, the activity does not appear equally increased (same average of downloads and uploads per member during these years, a slight increase in the number of active members) and a considerable number of members very rarely access the platform. We observed a paradoxical contrast between the increasing interest on this instrument (exponential increase of subscription to platform for headaches) and its consequent poor use by the registered specialists. There is no difference in its use between headache specialists and other specialists. Furthermore, on average the number of uploads is remarkably lower than downloads, indicating passive use of the platform.

In the other platforms, discussions and downloads are considerably less than the number of subscribers, similarly to the activity of our platform. These results confirm the potential but also the limits of digital instruments in the training for specialists, both in headaches and in other disorders.

In order to improve the functionality of our platform, a total renewal of the graphic interface is already in place, which will make it even easier and more pleasant to use. Future perspectives could be the use of typical tools of social networks such as the possibility of writing posts even without uploading content and adding reactions to the interventions of other users, the creation of a chat that allows the creation of live discussion groups or the interaction with other platforms.

**References**

1. Raieli V, Correnti E, Sandullo A, Romano M, Marchese F, Loiacono C, Brighina .F.

Effectiveness of a digital platform for sharing knowledge on headache management: a two- year experience. Funct Neurol. 201833(1):51-55;

2. Raieli V, Loiacono C, Messina LM, Correnti E, Brighina F. Is a digital platform useful in headache training? A 4-year Italian experience. Neurol Sci.2018 ,39.2223-2224;

3. Raieli V. , Messina L.M. , Vetri L., Drago F. Brighina F. The Utility of a Digital Platform to Help Specialists Training in The Management of Headaches: An Italian Experience. Biomedical Journal of Scientific and Techinical Research. 2019 ,15(1) pp.1-4

**Conflict of interest**: none 
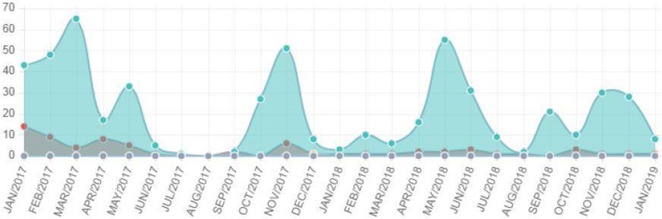


## A15 Placebo And Nocebo Responses In Anti-CGRP Monoclonal Antibody Trials For Migraine Prevention: a meta-analysis

### Kokoti L^3,*^, Drellia K^4,*^, Papadopoulos D^2^, Mitsikostas DD^1^

#### ^1^1st Neurology Department, Aeginition Hospital, Medical School, National and Kapodistrian University of Athens, Athens 11528-GR, Greece; ^2^Neurology Clinic, Athens Medical Center-Paleo Phaliro Clinic, 36 Areos street, Paleo Phaliro Athens 17562-GR, Greece; ^3^1st Neurology Department, Aeginition Hospital, Medical School, National and Kapodistrian University of Athens, Athens 11528-GR, Greece; ^4^1st Neurology Department, Aeginition Hospital, Medical School, National and Kapodistrian University of Athens, Athens 11528-GR, Greece

##### **Correspondence:** Drellia K(konstdrl@gmail.com)

^*^equal contribution

**Abstract**

**Βackground:** Placebo is the therapeutic response after the administration of a pharmacologically inactive agent. Its negative equivalent, nocebo refers to the development of adverse events (AEs) from the administration of an inert substance and it is very prevalent among treatments for neurological disorders, resulting in low adherence and poor treatment outcome.

**Objective**: To estimate the incidence of placebo and the incidence and severity of nocebo responses in clinical trials of monoclonal antibodies against the calcitonin gene-related peptide (CGRP) pathway for episodic (EM) and chronic migraine (CM) and compare them to those of topiramate and onabotulinum toxin A as standard treatments for EM and CM, respectively.

**Methods**: We conducted a systematic Pubmed and clinicaltrials.gov search for all randomised, placebo controlled, phase 3 trials of anti-CGRP monoclonals, topiramate and onabotulinum toxin A. A meta analysis of the frequency of placebo effect was performed by pooling the percentage of placebo-treated patients who had at least 50% reduction from baseline in migraine days per month. Meta- analysis of the incidence of nocebo responses was performed by pooling the percentage of placebo-treated patients that exhibited any adverse event. Nocebo severity was calculated from the percentage of placebo-treated patients that exhibited any serious adverse events (SAE) and the percentage of placebo-treated patients that dropped-out due to AEs.

**Results**: The pooled estimate for placebo frequency was 30% (95% CI: 24.1 - 36) in EM trials and 24.1% (95% CI: 11.1 - 37.2) in CM trials of anti-CGRP monoclonal, whereas the pooled placebo frequency in topiramate trials in EM was estimated to be 24.2% (95% CI: 19.5 - 28.8).

The pooled estimate of incidence of nocebo responses was 59.6% (95% CI: 57.2 - 62) in anti-CGRP trials for EM. The pooled serious nocebo responses and the pooled estimate of drop-outs due to nocebo were 1.5% (95% CI: 1.0 – 2.0) and 1.5% (95% CI: 0.7 - 2.2), respectively in anti CGRP trials for EM. On the other hand, the pooled incidence of dropouts due to nocebo in topiramate trials in EM was 9.2% (95% CI: 7 – 11.5).

The pooled estimate of incidence of nocebo responses in CM trials of anti-CGRP agents was 51.1% (95% CI: 37.9 – 64.4) and the pooled serious nocebo responses and the pooled estimate of drop-outs due to nocebo were 0.9% (95% CI: 0.4 - 1.4) and 1.1% (95% CI: 0.3 – 1.8), respectively. In onabotulinum toxin A trials in CM, the overall nocebo frequency was 51.6% (95% CI: 42.1-61.1), the pooled serious nocebo response and drop-out frequency due to nocebo were 2.3% (95%CI: 1.2 -3.4) and 0.7% (95%CI: 0.1 – 1.3).

**Conclusions:**

Placebo responses in anti-CGRP monoclonal trials are average and comparable to those seen in topiramate trials. Nocebo responses in trials of anti-CGRP monoclonals for EM, were average or low, compared to treatments of other neurological disorders and were lower than those seen in topiramate trials for EM. Nocebo responses in trials of anti-CGRP monoclonals in CM, were average or low and comparable to those seen in onabotulinum toxin A trials for CM.

## A16 Functional connectivity and photo-, phono-, osmophobia in migraine

### Noboru Imai^1^, Asami Moriya^1^, Eiji Kitamura^2^

#### ^1^Department of Neurology, Japanese Red Cross Shizuoka Hospital, Shizuoka, Shizuoka, Japan, ^2^Department of Neurology, Kitasato University, Sagamihara, Kanagawa, Japan

##### **Correspondence:** Noboru Imai(neurologyimai@gmail.com)

**Background:** Migraine attacks are typically associated with photo-, phono-, or osmophobia. To explore the aforementioned 3 conditions in migraine development, we investigated differences in whole brain resting-state functional connectivity (FC) between patients with and without these conditions. **Methods:** Sixty-two women with migraine underwent resting-state functional magnetic resonance imaging during the interictal phase. We compared resting-state FC between subjects with and without photo-, phono-, or osmophobia using region of interest to region of interest analysis of 91 cortical, 15 subcortical, and 26 cerebellar areas. **Results:** The patients with photophobia showed a higher connectivity between the vermis 9 (ver9) and bilateral (bil) intracalcarine cortex (IC)/bil cuneal cortex (cuneal)/bil lingual gyrus (LG)/bil supracalcarine cortex, as well as the right (r) cerebellum (cereb) 4-6 between the r cuneal and left (l) amygdala/l accumbens/r paracingulate gyrus. Photophobia patients also demonstrated higher connectivity between the l cuneal and l amygdala, as well as a lower connectivity between the ver9 and l angular gyrus than did patients without photophobia. The patients with phonophobia demonstrated a higher connectivity between the l cereb8-9 and bil cereb6/r temporal occipital fusiform cortex (TOFusC), between the ver9 and bil cereb6/l TOFusC/l inferior temporal gyrus/l superior temporal gyrus, between the r IC and l supramarginal gyrus, and between the l amygdala and l parahippocampal gyrus, as well as a lower connectivity between the cereb7-8 and r middle frontal gyrus/r aTOFusC than did patients without phonophobia. Finally, the patients with osmophobia demonstrated a higher connectivity between r cereb6 and the brainstem than did those without osmophobia. **Discussion:** Our results suggest that the cerebellum plays a key role in the relationship between photo-, phono-, and/or osmophobia and neural FC, particularly in migraine development.

## A17 ANTI-CALCITONIN GENE-RELATED PEPTIDE MONOCLONAL ANTIBODIES: ADVERSE EFFECTS. WHERE DO WE STAND?

### Theodoros Mavridis, Chrysoula Koniari, Breza Marianthi, Nikolaos Fakas and Dimos D. Mitsikostas

#### ^1^1st Neurology Department, Aeginition Hospital, School of Medicine, National and Kapodistrian University of Athens, Athens, Greece

##### **Correspondence:** Theodoros Mavridis(mavridismdr@gmail.com)

**Background**

The discovery of anti-calcitonin gene-related peptide (anti-CGRP) and its connection to migraine, led to the development of disease-specific drugs. The four novel anti-CGRP monoclonal antibodies (mAb) have been investigated in a large Phase II–III clinical programme, showing similar efficacy to the currently used drugs for migraine prevention but a lot of skepticism exists regarding their adverse events.

**Methods**

Literature searches were conducted on MEDLINE (PubMed), Scopus, and clinicaltrials.gov, using several key words and their combinations. Search terms included: migraine, anti-calcitonin gene-related peptide monoclonal antibodies, adverse events. References from the selected articles were also thoroughly screened for other pertinent articles. During the screening of the abstracts/full texts, the publications that were not relevant to this review were removed**.**

**Results**

The most common adverse events that have been reported more frequently in the active anti-CGRP mAb arms versus placebo arms are injection-site pain, erythema, upper respiratory infection, nasopharyngitis, fatigue, influenza, joint pain, back pain and headache. The main concern regarding their use was related to the potential cardiovascular effects and liver toxicity. Existing data do not confirm any cardiovascular effect. No interactions with other preventative drugs have been reported, as mAb elimination is mainly the result of proteolysis and does not involve metabolism by liver enzymes, making drug–drug interactions and hepatotoxicity unlikely.

**Discussion**

To date, the overall safety profile of anti-CGRP mAb for the prevention of migraine has been reported to be more than satisfactory. Safety questions were raised due to preclinical data from studying and blocking CGRP, but no safety flags occurred during development. Long-term Phase IV trials are needed to further evaluate the safety of anti-CGRP mAb.

## A18 A real-world analysis of the burden of migraine in patients with prior treatment failures: evidence from the BECOME study

### Christian Lucas^1^, Patricia Pozo-Rosich^2^, David Watson^3^, Charly Gaul^4^, Emma Ramsden^5^, Shannon Ritter^6^, Paolo Martelletti^7^, Josefin Snellman^5^

#### ^1^Pain Clinic, Service de Neurochirurgie, Hôpital Salengro, CHRU de Lille, Lille Cedex, France; ^2^Headache Unit, Neurology Department, Vall d'Hebron University Hospital, Barcelona, and Headache and Neurological Pain Research Group, Vall d’Hebron Institute of Research (VHIR), Universitat Autònoma de Barcelona, Barcelona, Spain; ^3^Hamilton Medical Group, Aberdeen, Scotland; ^4^Migraine and Headache Clinic Königstein, Königstein im Taunus, Germany; ^5^Novartis Pharma AG, Basel, Switzerland; ^6^Novartis Pharmaceuticals Corporation, East Hanover, NJ, USA; ^7^Department of Clinical and Molecular Medicine, Sapienza University of Rome, Sant'Andrea Hospital, Via di Grottarossa, Rome, Italy

##### **Correspondence:** Patricia Pozo-Rosich(ppozorosich@yahoo.com)

**BACKGROUND**

Limited European data are available on the burden of disease and quality of life (QoL) in migraine patients with prior prophylactic treatment failures (PPTF). The aim of this analysis was to characterise the burden of migraine in patients with≥1 PPTF and ≥4 monthly migraine days (MMD).

**METHODS**

BECOME was a prospective, multicentre, non-interventional study in adult patients with migraine, conducted in two concurrent parts over 3 months across Europe and Israel. Part 1 assessed the characteristics of all migraine patients visiting headache specialist centres over a 3-month prospective period, including frequency of PPTF (results presented separately). Part 2 of the study examined burden of disease using patient-reported outcome questionnaires (**Table 1**) in visiting migraine patients with ≥1 PPTF and ≥4 MMD, identified by study investigators during Part 1.

**RESULTS**

Overall, 20,837 patients were screened in Part 1; 2419 were included in the Part 2 analysis. The mean±SD age of patients in Part 2 was 43.0±11.56 years; the majority were female (86.9%) and diagnosed with migraine without aura (53.4%). Overall, Part 2 patients reported a EuroQol visual analogue scale (VAS) score of 67.3±20.36, severe impact (HIT-6 score 65.2±5.59), and severe disability (MIDAS disability grade IV, 81.9% of patients) due to headache (**Table 1**). The burden of disease generally increased in line with increases in PPTF and MMD (**Table 2**).

**DISCUSSION**

BECOME confirms the significant burden of disease among migraine patients who have failed prior prophylactic treatments and provides real-world evidence of the continuous need for improved treatment of patients with difficult-to-treat migraine.

**Abstract topic:** Epidemiology/Clinical Features

**Conflicts of Interest:** This study was funded by Novartis Pharma AG, Basel, Switzerland. **Christian Lucas** — collaboration as expert, investigator or coordinator of clinical trials with Novartis, Teva, Sanofi, Grunenthal, Eli Lilly, Biogen, Ethypharm. **Patricia Pozo-Rosich** — received research grants from Allergan and Novartis, and consulting and educational fees from Allergan, Almirall, Chiesi, Eli Lilly, Novartis and Teva. **David Watson** — honoraria from Novartis and Teva and Allergan in last 12 months for consultancy work and educational work. **Charly Gaul** — received honoraria for consulting and lectures within the past three years from Allergan Pharma, Ratiopharm, Boehringer Ingelheim Pharma, Eli Lilly, Novartis Pharma, Desitin Arzneimittel, Cerbotec, Bayer vital, Hormosan Pharma, electroCore, Grünenthal, Reckitt Benckiser, and Teva. He does not hold any stocks of pharmaceutical companies or medical device companies. **Emma Ramsden** — Provides services to Novartis Pharma AG. **Paolo Martelletti** — honoraria/expenses, consulting/advisor boards, funded research: Allergan, Amgen, Electrocore, Eli Lilly, Novartis, Springer HealthCare, Teva. **Shannon Ritter**, and **Josefin Snellman** — employees and stocks: Novartis.

**Table 1 (Abstract A18).** Characteristics and PRO scores of the BECOME study population set for Part 2

	Part 2 (N=2419)
Age, years	43.0 (11.56)
Female, n (%)	2103 (86.9)
Time from the first diagnosis of migraine, years	15.7 (11.92)
Type of migraine*, n (%)	
Migraine without aura	1291 (53.4)
Migraine with aura	290 (12.0)
Chronic migraine	731 (30.2)
Complications of migraine	9 (0.4)
Probable migraine	2 (0.1)
Episodic syndromes that may be associated with migraine^#^	6 (0.2)
Diagnosis of medication overuse headache, n (%)	571 (23.6)
PRO questionnaires	
EQ-5D-5L utility index score	0.76 (0.22)
EQ VAS score	67.3 (20.36)
HIT-6 total score	65.2 (5.59)
MIDAS disability grade IV, n (%)	1982 (81.9)

All values are mean (SD), unless otherwise indicated. *diagnosis according to the ICHD 3-beta code; ^#^previously known as ‘childhood periodic syndromes’, this type of migraine may also occur in adults, who also have migraine with or without aura, or who have an increased likelihood to develop either of these disorders. EQ-5D-5L, EuroQol 5 dimensions 5 levels; HIT, Headache Impact Test; ICHD, International Classification of Headache Disorders; MIDAS, migraine disability assessment; N, total number of patients; n, number of patients; PRO, patient-reported outcomes; SD, standard deviation; VAS, visual analogue scale

**Table 2 (Abstract A18).** PRO scores of the BECOME study population set for Part 2 by PPTF and MMD

PRO questionnaires	Part 2 (N=2419)			
	1 PPTF (n=1034)	2 PPTF(n=690)	3 PPTF (n=324)	≥4 PPTF (n=371)
EQ-5D-5L utility index score	0.78 (0.22)	0.76 (0.22)	0.72 (0.24)	0.72 (0.22)
EQ VAS score	70.3 (19.85)	66.8 (20.07)	64.5 (20.31)	62.0 (20.94)
HIT-6 total score	64.6 (5.74)	65.1 (5.69)	66.4 (5.03)	66.3 (5.14)
MIDAS disability grade IV, n (%)	802 (77.6)	565 (81.9)	283 (87.3)	332 (89.5)
	4–7 MMD(n=806)	8–14 MMD(n=605)	≥15 headache days/month, of which ≥8 are migraine days (n=1007)	
EQ-5D-5L utility index score	0.82 (0.19)	0.81 (0.19)	0.68 (0.24)	
EQ VAS score	73.7 (17.88)	72.6 (17.74)	59.0 (20.71)	
HIT-6 total score	63.6 (5.89)	65.0 (5.41)	66.7 (5.04)	
MIDAS disability grade IV, n (%)	583 (72.3)	492 (81.3)	907 (90.1)	

All values are mean (SD) unless otherwise indicated. EQ-5D-5L, EuroQol 5 dimensions 5 levels; HIT, Headache Impact Test; MIDAS, migraine disability assessment; MMD, monthly migraine days; N, total number of patients; n, number of patients; PPTF, prior prophylactic treatment failures; PRO, patient-reported outcomes; VAS, visual analogue scale

## A19 Characteristics of migraine patients visiting the European headache specialist centres: real-world evidence from the multinational BECOME study

### Patricia Pozo-Rosich^1^, Christian Lucas^2^, David Watson^3^, Charly Gaul^4^, Emma Ramsden^5^, Shannon Ritter^6^, Paolo Martelletti^7^, Josefin Snellman^5^

#### ^1^Headache Unit, Neurology Department, Vall d'Hebron University Hospital, Barcelona, and Headache and Neurological Pain Research Group, Vall d’Hebron Institute of Research (VHIR), Universitat Autònoma de Barcelona, Barcelona, Spain; ^2^Pain Clinic, Service de Neurochirurgie, Hôpital Salengro, CHRU de Lille, Lille Cedex, France; ^3^Hamilton Medical Group, Aberdeen, Scotland; ^4^Migraine and Headache Clinic Königstein, Königstein im Taunus, Germany; ^5^Novartis Pharma AG, Basel, Switzerland; ^6^Novartis Pharmaceuticals Corporation, East Hanover, NJ, USA; ^7^Department of Clinical and Molecular Medicine, Sapienza University of Rome, Sant'Andrea Hospital, Via di Grottarossa, Rome, Italy

##### **Correspondence:** Patricia Pozo-Rosich(ppozorosich@yahoo.com)

**BACKGROUND AND AIMS**

Real-world evidence on the characteristics of patients with migraine from Europe is limited. The current study provides real-world evidence on the characteristics of patients visiting European headache specialist centres.

**METHODS**

BECOME was a prospective, multicentre, non-interventional study in adult patients (18–65 years) with migraine consisting of two parts, conducted across Europe and Israel. In Part 1, all patients visiting the participating headache specialist centres over a 3-month prospective period were screened for frequency of prior prophylactic treatment failures (PPTF), monthly migraine days (MMD), and other characteristics (**Table 1**). Patients identified by study investigators with ≥1 PPTF and ≥4 MMD were enrolled in Part 2 of the study, which examined the burden of disease and healthcare resource utilisation (results presented separately).

**RESULTS**

Of the 163 centres participating in the study, 156 had data available for Part 1. As shown in **Table 1**, 20,837 patients with migraine visited the headache centres during screening period, of which 62.2% reported ≥1 PPTF and 74.3% reported ≥4 MMD. Furthermore, 16.9% of patients reported ≥4 PPTF and 22.9% reported ≥15 headache days with ≥8 MMD. Approximately a quarter (27.0%) of patients with migraine visited the centres for the first time.

**DISCUSSION**

The BECOME study represents real-world characteristics of the migraine patient population visiting headache specialist centres in Europe and Israel. Nearly 17% of the study population reported ≥4 prior prophylactic treatment failures and more than 20% reported ≥15 headache days per month, demonstrating the high burden of disease, and a high unmet need with current prophylactic migraine therapy.

**Abstract topic:** Epidemiology/Clinical Features

**Conflicts of Interest:** This study was funded by Novartis Pharma AG, Basel, Switzerland. **Patricia Pozo-Rosich** — received research grants from Allergan and Novartis, and consulting and educational fees from Allergan, Almirall, Chiesi, Eli Lilly, Novartis and Teva. **Christian Lucas** — collaboration as expert, investigator or coordinator of clinical trials with Novartis, Teva, Sanofi, Grunenthal, Eli Lilly, Biogen, Ethypharm. **David Watson** — honoraria from Novartis and Teva and Allergan in last 12 months for consultancy work and educational work. **Charly Gaul** — received honoraria for consulting and lectures within the past three years from Allergan Pharma, Ratiopharm, Boehringer Ingelheim Pharma, Eli Lilly, Novartis Pharma, Desitin Arzneimittel, Cerbotec, Bayer vital, Hormosan Pharma, electroCore, Grünenthal, Reckitt Benckiser, and Teva. He does not hold any stocks of pharmaceutical companies or medical device companies. **Emma Ramsden** — Provides services to Novartis Pharma AG. **Paolo Martelletti** — honoraria/expenses, consulting/advisor boards, funded research: Allergan, Amgen, Electrocore, Eli Lilly, Novartis, Springer HealthCare, Teva. **Shannon Ritter**, and **Josefin Snellman** — employees and stocks: Novartis.

**Table 1 (Abstract A19).** Characteristics of the BECOME study population in Part 1

	Part 1 (N=20,837)
Prior prophylactic treatment failures	
No PPTF	7880 (37.8)
≥1 PPTF	12,957 (62.2)
1 PPTF	4137 (19.9)
2 PPTF	3063 (14.7)
3 PPTF	2244 (10.8)
≥4 PPTF	3513 (16.9)
Monthly migraine days	
<4 MMD	5358 (25.7)
≥4 MMD	15,479 (74.3)
4–7 MMD	5796 (27.8)
8–14 MMD	4916 (23.6)
≥15 headache days, of which ≥8 were MMD	4767 (22.9)
First visit versus follow-up visit patients	
First visit	5621 (27.0)
Follow-up visit	15,216 (73.0)
Medication overuse	
Any medication overuse	3706 (17.8)
Any suspected medication overuse headache	2464 (11.8)
Outpatients versus inpatients	
Outpatient visits	19,700 (94.5)
Inpatient visits	650 (3.1)
Unclassified visits	487 (2.3)

All values are n (%) of patients

MMD, monthly migraine days; N, total number of patients; n, number of patients; PPTF, prior prophylactic treatment failures

## A20 Short and Mid-term predictors of response to OnabotulinumtoxinA: a practical approach from a Spanish Headache Specialized Unit

### A.Alpuente^1,2^, VJ. Gallardo^2^, M.Torres-Ferrús^1,2^, J.Álvarez-Sabín^1^, P.Pozo-Rosich^1^

#### ^1^Headache Unit, Neurology Department, Hospital Universitari Vall d'Hebron, Barcelona, Spain; ^2^Headache Research Group, Vall d'Hebron Research Institute (VHIR), Universitat Autònoma de Barcelona, Barcelona, Spain

##### **Correspondence:** VJ. Gallardo(victor.gallardo@vhir.org)

**Background:** To identify clinical predictors of response to OnabotulinumtoxinA in patients with chronic migraine (CM) at 6 and 12 months of follow-up.

**Methods:** A three-year prospective observational study. We included patients diagnosed with CM. We collected clinical data and treatment response variables. Patients were classified according to their improvement in frequency: poor (<25%), good (≥50%) and excellent (≥75%) responders. A comparative analysis was carried out at 6 and 12 months identifying clinical predictors of treatment response in each timepoint.

**Results:** Data was collected from 221 patients (86.1% women, mean age 45.9±11.3 years and mean migraine evolution time 27.6±14.3 years). A 54.2% had medication overuse (MO), 27.5% with aura and a 70.3% presented anxiety. After 12 months, we observed a significant mean reduction in frequency (from 24.3±7.0 to 13.9±9.6 d/mo) and analgesic medication use (from 38.1±30.4 to 17.5±13.5 p/mo). At month 6, excellent response was associated with daily frequency patients with MO (p<0.01) and higher migraine days/month rate (p<0.05). At month 12, clinical predictors of excellent response were patients with less years of migraine evolution (p<0.01) and the presence of anxiety and aura (p<0.05). Moreover, excellent responders showed a higher improvement rate in pain intensity at 6 and 12 months than the other responder groups.

**Discussion:** In our cohort, we showed that predictors of excellent response to OnabotulinumtoxinA is different at each timepoint of treatment. Daily frequency patients with MO will only need 6 months of treatment to clinically improve. Patients with comorbidities who start treatment earlier in the course of the disease will need longer treatment duration.

## A21 Backstage Hero of Chronicity in Migraine Naturel History: Co morbidity

### Ayşe Begüm Büyüksural ^1^, Aynur Özge^1^, Didem Derici Yıldırım ^2^, Bahar Taşdelen^2^

#### ^1^Mersin University Faculty of Medicine, Department of Neurology, Mersin, Turkey; ^2^Mersin University Faculty of Medicine, Department of Biostatistic, Mersin, Turkey

##### **Correspondence:** Aynur Özge(aynurozge@gmail.com)

**Main objective:** There are restricted evidence based data about the chronification of migraine in the natural course of the disease. The main objective of this study was to determine the factors affecting the chronicity of patients with episodic migraine in patients undergoing headache outpatient process.

**Patients and Methods:** This study is conducted as a part of Turkish Headache Database Project. 3629 migraine patients registered in database, between 2000-2017. Among these, 2042 of these patients were diagnosed as episodic migraine and 1227 had chronic migraine at first visit. 68 patients undergoing follow-up at least two visits, transformed chronic migraine and compared to others. Demographic, clinic, phenotypic, epigenetic factors were compared between chronics and other patients. Statistical analysis was performed accordingly.

**Results:** The results showed there is a statistically significant relationship between chronicity and vertigo (p=0.007), cranial autonomic symptoms (p=0.037) and allergy (p=0.026). Co morbidity of allergy in migraine patients with chronic is 7.624 times higher than the episodic migraines. In addition, osmophobia (p=0.017), comorbid concomitant Diabetis Mellitus (p=0.016), coronary artery disease (p=0.025) and headache triggered by hunger (p=0.001) were observed to be more prevalent in episodic migraine.

**Conclusion:** The study results showed that the presence of vertigo, cranial autonomic symptoms, concomitant allergy were effective in the chronicity of migraine with requires much more attention to migraine comorbidities associated rather than diagnostic peculiarities, not only for diagnosis but also for defining coping options.

## A22 Long-term efficacy and safety of erenumab: Results from 64 weeks of the LIBERTY study

### Uwe Reuter^1^, Peter J. Goadsby^2^, Michel Lanteri-Minet^3,4^, Peggy Hours-Zesiger^5^, Chrystel Fernandes^5^, Nadia Tenenbaum^6^, Michel D. Ferrari^7^, Jan Klatt^5^

#### ^1^Department of Neurology, Charité Universitätsmedizin Berlin, Berlin, Germany; ^2^NIHR-Wellcome Trust, King’s Clinical Research Facility, King’s College London, London, UK; ^3^Pain Department, CHU Nice, Nice, France; FHU InovPain; ^4^Université Côte d'Azur, Nice, France; ^5^Novartis Pharma AG, Basel, Switzerland; ^6^Novartis Pharmaceutical Corporation, East Hanover, NJ, USA; ^7^Department of Neurology, Leiden University Medical Center, Leiden, the Netherlands

##### **Correspondence:** Uwe Reuter(uwe.reuter@charite.de)

**Background**

The LIBERTY study (NCT03096834) demonstrated efficacy of erenumab 140 mg in episodic migraine patients with 2-4 prior preventive treatment failures. The aim of this analysis was to assess efficacy and safety of erenumab at Week 64 of the LIBERTY study.

**Methods**

Patients completing the 12-week double-blind treatment phase (DBTP) of the LIBERTY study (N=240) initially randomised to placebo and erenumab 140 mg subcutaneous injections were enrolled into the open-label extension phase (OLEP) to receive open-label treatment with monthly erenumab 140 mg for 3 years. The efficacy outcomes included ≥50%/≥75%/100% reduction from the DBTP baseline in monthly migraine days (MMD) (responder rates), change from the DBTP baseline in MMD, Headache Impact Test total score, and Migraine Physical Function Impact Diary (Everyday Activities and Physical Impairment) scores in the overall population, patients on continuous erenumab and patients from placebo group who initiated erenumab. The outcomes were assessed throughout the first 52 weeks in OLEP (total 64 weeks from DBTP baseline).

**Results**

Overall, 204/240 (85.0%) patients completed the Week 52 visit of the OLEP. Results for the outcomes measured are presented in **Table 1**. On all efficacy outcomes assessed, patients on continuous erenumab showed sustained efficacy, while those who initiated erenumab in the OLEP showed sustained improvement from Week 13 onwards. Nearly 80.8% (overall group), 76.3% (continuing erenumab) and 85.2% (initiating erenumab) of patients reported adverse events (AEs). The corresponding incidences for serious AEs were 6.7%, 5.9%, and 7.4%. No deaths were reported.

**Discussion**

Efficacy of erenumab was sustained throughout 64 weeks in a difficult-to-treat patient population with multiple prior preventive treatment failures in both continuous erenumab and those initiating erenumab treatment groups. Safety of erenumab was in line with previously reported clinical trials.

**Character count**: 1948 characters with spaces excluding table.

**Abstract topic**: Treatments

**Conflicts of interest**: The study was funded by Novartis Pharma AG, Basel, Switzerland. Erenumab is co-developed by Novartis and Amgen.

**Uwe Reuter** — received consulting fees, speaking/teaching fees, from Allergan, Amgen, Autonomic Technologies, CoLucid, ElectroCore, EliLilly, Medscape, Novartis, StreamMedUp, TEVA Pharmaceuticals and research grants from Allergan, Amgen, Autonomic Technologies, CoLucid, ElectroCore, EliLilly, Medscape, Novartis, StreamMedUp, TEVA Pharmaceuticals. **Peter J Goadsby** — received personal fees from Amgen and Eli-Lilly and Company, Alder Biopharmaceuticals, Allergan, Autonomic Technologies Inc., Dr Reddy’s Laboratories, Electrocore LLC, eNeura, Novartis, Scion, Teva Pharmaceuticals, and Trigemina Inc., MedicoLegal work, Journal Watch, Up-to-Date, Oxford University Press, Massachusetts Medical Society, and Wolters Kluwer and grants from Amgen and Eli-Lilly and Company, Alder Biopharmaceuticals, Allergan, Autonomic Technologies Inc., Dr Reddy’s Laboratories, Electrocore LLC, eNeura, Novartis, Scion, Teva Pharmaceuticals, and Trigemina Inc. **Michel Lanteri-Minet** — received honoraria for advisory boards, speaker panels or investigation studies from Allergan, Amgen, Astellas, ATI, BMS, Boehringer, Boston Scientific, CoLucid, Convergence, Glaxo-SmithKline, Grunenthal, Lilly, Medtronic, Menarini, MSD, Novartis, Pfizer, Reckitt Benckiser, Saint-Jude, Sanofi-Aventis, Teva, UCB, Zambon. **Peggy Hours-Zesiger, Nadia Tenenbaum** and **Jan Klatt** — employees and stocks: Novartis.

**Chrystel Fernandes** — employee: Novartis. **Michel Ferrari** — consultancy from Medtronic, Electrocore, Amgen, Lilly, Teva, and Novartis, and independent support from the European Community, NWO, NIH and the Dutch Heart Foundation and grants, trial support from Medtronic, Electrocore, Amgen, Lilly, Teva, and Novartis, and independent support from the European Community, NWO, NIH and the Dutch Heart Foundation.

**Table 1 (Abstract A22).** Efficacy outcomes measures at the end of the first year of the OLEP, Observed (Open-Label Analysis Set)

Outcomes	Values at Week 64 (Week 52 of OLEP)		
	Patients on erenumab 140 mg continued on erenumab 140 mg in the OLEP, N=118	Patients on placebo who initiated erenumab 140 mg in the OLEP, N=122	Overall population, N=240
≥50% reduction in MMD	44.3%	50.0%	47.1%
≥75% reduction in MMD	21.4%	25.8%	23.5%
100% reduction in MMD	8.6%	16.7%	12.5%
Change from the DBTP baseline in MMD	−3.8 (3.9)	−3.6 (4.4)	−3.7 (4.1)
Change from the DBTP baseline in HIT-6	−8.5 (7.4)	−9.7 (10.0)	−9.0 (8.7)
Change from the DBTP baseline in MPFID-PI	−5.2 (6.9)	−4.5 (8.4)	−4.8 (7.7)
Change from the DBTP baseline in MPFID-EA	−6.6 (7.7)	−5.1 (9.4)	−5.9 (8.6)

Data are mean (SD) or % of the patients with non-missing value at Week 64; Data for HIT-6 reported at Week 60.

DBTP, double-blind treatment phase; HIT-6, Headache Impact Test; MMD, monthly migraine days; MPFID-EA; Migraine Physical Function Impact Diary-everyday activities MPFID-PI, Migraine Physical Function Impact Diary-physical impairment; N, number of subjects included in the analysis set; OLEP, open-label extension phase; SD, standard deviation

## A23 IMPACT OF MEDICAL CARE ON SYMPTOMATIC DRUG CONSUMPTION AND QUALITY OF LIFE IN HEADACHE PATIENTS : a one year population study.

### Antonaci F^1^, Cotta Ramusino M^1^, Vanacore N^2^, Costa A^1^

#### ^1^Department of Brain and Behavioral Sciences, C. Mondino National Institute of Neurology Foundation, IRCCS, University of Pavia, Pavia, Italy; ^2^National Centre for Epidemiology, Surveillance, and Health Promotion, National Institute of Health, Rome, Italy.

##### **Correspondence:** Antonaci F(antonaci@unipv.it)

**Background:** Headache is one of the most common painful syndrome and can be responsible for high disability. It is a widespread disorder both in episodic and chronic form. Chronic headache often leads to a high use/overuse of symptomatic drugs; indeed, medication overuse headache (MOH) occurs in over half of chronic headache patients, with significant management difficulties.

**Objective:** to provide data about symptomatic drug (NSAIDs and triptans) consumption in an outpatient population of the Health District of Pavia, and describe how the clinical picture may change after being taken over by headache experts.

Materials and Methods: 276 patients using symptomatic drug for headache were recruited in 32 pharmacies. A telephonic interview was carried out in 199 of them. Data collection included sociodemographic characteristics and features of headache and drug consumption/abuse. Patients underwent 4 visits: a baseline visit (T0) and 3 follow-up visits performed by a neurologist at 3, 6 and 12 months (T3, T6 and T12, respectively). During each visit, patients underwent a complete neurological assessment and received therapeutic adjustments aimed at obtaining a proper management of headache.

**Results:** Patients with chronic migraine or MOH were 16% and 12%, respectively, at the telephonic interview. After 12 months of follow-up, we observed a significant decrease in the frequency of attacks (T0: 9±9/month vs T12: 2±2/month; p<0.001), in the days/month of headache (T0: 11±9 vs T12: 4±4; p<0.001), and in the duration of the single attack (T0: 34±30 hrs vs T12: 10±19 hrs; p<0.001). The improvement of headache management resulted in both a significant decrease in the analgesic consumption per month (T0: 12±16 vs T12: 4±6 doses/month; p=0.014), and an increase in the quality of life, scored by MIDAS and HURT (p<0.001).

**Conclusion:** This study shows that a proper medical management is more effective than self-treatment of headache, resulting in lower disability and improved quality of life within a few months from taking-over by headache specialists. RC MINSAL 2013-2015 IRCCS MONDINO

F. Antonaci MD PhD

Professor of Neurology

Mondino National Institute of Neurology Foundation,

Dept. of Brain and Behavioral Sciences

University of Pavia, Italy.

fabio.antonaci@unipv.it

www.fabioantonaci.it

## A24 Typical aura without headache: A case series of 5 patients

### Spanou Ioanna, Rizonaki Konstantina, Liakakis Georgios^,^ Bougea Anastasia, Xirou Sophia, Mitsikostas Dimos-Dimitrios, Kararizou Evangelia

#### 1^st^ Department of Neurology, Eginition Hospital, National and Kapodistrian University of Athens, Greece

##### **Correspondence:** Bougea Anastasia(annita139@yahoo.gr)

**Background**

Typical aura without headache (TAH) presents either as an isolated symptom in 4% of migraineurs or may occur typically later in life in 38% of migraineurs with headache. TAH describes typical aura neither accompanied nor followed by headache.

**Methods**

We present a case series of 5 patients with isolated TAH.

**Results**

**Case 1**: A 66-year-old man 8 years ago experienced recurrent visual episodes with gray waves from the periphery to the center bilaterally for 5-15min. Clinical and diagnostic work-up were unremarkable.

**Case 2**: A 34-year-old woman from a decade ago had recurrent episodes of blurred vision with flashes of light bilaterally, started peripherally and gradually progressed centrally for 15-30min. Clinical and diagnostic investigation were normal.

**Case 3**: A 37-year-old woman 7 years ago described episodes of visual blurring with small bright dots and flashes of light, often colorful, centrally that gradually progressed peripherally either in the left or in the right eye for 20-30min. Also, 2 years ago she had an episode with typical visual symptoms followed by numbness in her left face and arms for 30min. Brain magnetic resonance imaging (MRI) was normal and brain magnetic resonance angiography revealed a small aneurysm of the right anterior choroidal artery.

**Case 4**: A 49-year-old woman 5 years ago had recurrent episodes of bright waves in both temporal visual fields for 10 min. Neurological examination was normal, brain MRI had small non-enhancing lesions and neck vessels ultrasound was normal.

**Case 5:** A 51-year-old woman had brief visual episodes with bright, colorful shapes, small lights and bright zigzag lines from 1 year ago. Clinical examination and brain MRI were normal.

**Discussion**

TAH is a diagnosis of exclusion. Differential diagnosis from organic diseases, such as transient ischemic attack, seizure, subarachnoid hemorrhage and brain tumor is mandatory. Careful medical history combined with the appropriate diagnostic work-up are highly recommended.

**References**

1. Shah DR, Dilwali S, Friedman DI. Current Aura Without Headache. Current pain and headache reports. 2018;22(11):77.

Written, informed consent for publication was obtained from the patient.

## A25 The relationship between primary headaches and thyroid disorders: A retrospective study from a Greek Headache Outpatient Clinic

### Spanou Ioanna, Christidi Foteini, Liakakis Georgios, Rizonaki Konstantina, Bougea Anastasia, Anagnostou Evangelos, Kararizou Evangelia

#### 1^st^ Department of Neurology, Eginition Hospital, National and Kapodistrian University of Athens, Greece

##### **Correspondence:** Bougea Anastasia(annita139@yahoo.gr)

**Background**

Primary headaches and thyroid disorders constitute common medical conditions; with a prevalence of 15% for migraine, 38% for tension-type headache (TTH), and 0.2% - 5.3% for clinical hypothyroidism. To date, numerous studies suggested a bidirectional relationship between migraine and hypothyroidism, though certain had contradictory results.

**Methods**

We retrospectively evaluated the clinical records of 427 patients referred to the Headache Outpatient Clinic of Eginition Hospital from 2010 to 2018 and diagnosed with a primary headache. Thyroid dysfunction was assessed mostly based on patients’ self-reports.

**Results**

Out of 427 patients (Male/Female = 76 / 351), 253 patients (59.3%) were diagnosed with migraine without aura, 53 (12.4%) with TTH, 49 (11.5%) with migraine with aura, 29 (6.8%) with medication-overuse headache, 23 (5.4%) with mixed-type headache (migraine with/without aura and TTH), 9 (2.1%) with cluster headache, and 11 (2.6%) with other type of primary headache. The prevalence of any type of thyroid disorder was 20.8% (89/427 patients). In the total sample, 27 patients (6.3%) reported hypothyroidism, 12 (2.8%) Hashimoto’s thyroiditis, 3 (0.7%) hyperthyroidism, 18 (4.2%) unspecified thyroidopathy, 14 (3.3%) thyroid nodules, 3 (0.7%) thyroid goiter, and 12 (2.8%) thyroidectomy. Further statistical analysis between categorical variables (χ^2^) did not reveal any significant association (p>0.05) between headache subtypes and thyroid dysfunction, including specific categories.

**Discussion**

Thyroid disorder is frequent among primary headache patients. Although we did not find any specific association between headache subtypes and specific thyroid disorder, the high prevalence of thyroid dysfunction in general (20.7%) and specifically hypothyroidism (6.3%), needs further clarification in prospective longitudinal studies, highlights the importance of a multidisciplinary approach and might has significant clinical implications for patients’ treatment.

**References**

1. Steiner TJ, Stovner LJ, Katsarava Z, Lainez JM, Lampl C, Lanteri-Minet M, et al. The impact of headache in Europe: principal results of the Eurolight project. The journal of headache and pain. 2014;15:31.

2. Garmendia Madariaga A, Santos Palacios S, Guillen-Grima F, Galofre JC. The incidence and prevalence of thyroid dysfunction in Europe: a meta-analysis. The Journal of clinical endocrinology and metabolism. 2014;99(3):923-31.

3. Rainero I, Govone F, Gai A, Vacca A, Rubino E. Is Migraine Primarily a Metaboloendocrine Disorder? Current pain and headache reports. 2018;22(5):36.

## A26 Loss of consciousness and chronic post-traumatic headache

### Brad Torphy

#### Diamond Headache Clinic, Chicago, IL, USA Hayley Bemel, Argosy University, Chicago, IL, USA

##### **Correspondence:** Brad Torphy(torphymd@gmail.com)

**Background**

Nearly 1.7 million traumatic brain injuries (TBI) occur every year in the United States of America. Headache is the most common complaint, and it can occur after mild, moderate, or severe

injury. Several researchers have reported that post-traumatic headache is more common after mild TBI than after severe TBI - in patients who sustained a loss of consciousness. However, International Classification of Headache Disorders diagnostic criteria for post-traumatic headache attributed to mild head injury does not distinguish between loss of consciousness and no loss of consciousness, but rather include both “no loss of consciousness,” and “a loss of consciousness of < 30 minutes.” The purpose of this study was to determine if there was an association between loss of consciousness (regardless of duration) and the development of chronic post-traumatic headache.

**Methods**

A retrospective chart review was undertaken of new patients at a tertiary headache center with the diagnosis of chronic post-traumatic headache from 2013-2018. Physician documentation was examined for loss of consciousness at the time of the trauma. If the physician did not document loss of consciousness or maintenance of consciousness the patient was not included in this study.

**Results**

A total of 180 patient charts contained documentation regarding consciousness at the time of

the trauma. Loss of consciousness was reported in 68 cases, and no loss of consciousness was reported in 112 patients. We ran a Chi Square of the data using The Statistical Package for the Social Sciences to determine the asymptotic significance of 0.001. This result suggests that there is only a 0.1% chance of no relationship between loss of consciousness and chronic post- traumatic headache.

**Discussion**

In our study of patients with chronic post-traumatic headache, 62% of the patients did not lose consciousness during and after trauma compared with 38% who lost consciousness at the time of the trauma. There is an association between loss of consciousness and the development of chronic post-traumatic headache. In our study patients were more likely to develop chronic post- traumatic headache if there was no loss consciousness at the time of the trauma. This is consistent with most of the current literature that suggests mild TBI leads to post-traumatic headache more often than severe TBI. Given that mild TBI includes both “no loss of consciousness” and “loss of consciousness of < 30 minutes,” however, we believe that our research is novel because of the distinction between loss of consciousness and no loss of consciousness. This suggests that no loss of consciousness may be a risk factor for the development of chronic post-traumatic headache. It is likely that other factors, such as anxiety

or depression, may represent even greater risk for the development of this condition. Areas for future research include assessing the association of anxiety or depression, together with or without loss of consciousness and the development of chronic post-traumatic headache.

## A27 A possible role for 2-AG in the “addiction” aspect of medication‐overuse headache?

### Rosaria Greco^1^, Marta Allena^1^, Chiara Demartini^1^, Anna Maria Zanaboni^1,2^, Elena Tumelero^1^; Daniele Piomelli^3^, Roberto De Icco^1,2^, Natascia Ghiotto^1^, Grazia Sances^1^, Cristina Tassorelli^1,2^

#### ^1^Headache Science Centre, IRCCS Mondino Foundation, Pavia, Italy; ^2^Department of Brain and Behavioral Sciences, University of Pavia, Pavia, Italy; ^3^Deptartment of Anatomy and Neurobiology, University of California, USA

##### **Correspondence:** Marta Allena(marta.allena@mondino.it)

**Background**

The biological mechanisms of the progression of migraine from episodic to chronic are poorly understood. Medication overuse (MO) is considered by many as one of the main determinants of migraine progression. Recently, in a preliminary study, we have shown alterations of anandamide metabolism in circulating peripheral blood mononuclear cells (PBMCs) of migraineurs with a gradient of gravity from the episodic to the chronic type.

In this study we further investigated the asset of the endocannabinoid system (ES) in migraine as a function of disease severity.

**Methods**

Using rt-PCR we essayed the gene expression of the enzymes involved in the metabolism of 2-Arachidonoylglycerol (2-AG), sn-1-specific diacylglycerol lipase (DAGL) for the synthesis and monoacylglycerol lipase (MAGL) for the degradation, in the PBMCs of 21 subjects with episodic migraine (EM), 19 subjects with chronic migraine and MO (CM-MO) and an age- and sex-matched control group (CT, N=21). Subjects in CM-MO group were tested at baseline and 2 months after detoxification.

**Results**

The levels of MAGL mRNA were significantly higher both in the EM and CM-MO groups as compared with CT. DAGL gene expression was increased in CM-MO group at baseline and it decreased following detoxification, when it reached mRNA levels comparable to the EM group.

**Discussion**

These findings confirm the involvement of ES in migraine disease showing that not only anandamide, but also 2-AG metabolism is altered. In the case of CM-MO, our data point to an increased turnover of this latter endocannabinoid, a condition that is partially reverted by detoxification.

Authors declare no conflicts of interest.

## A28 Clinical Characteristics of Hemiplegic Migraine: a clinical study of 11 cases in Japan

### Daisuke Danno^1^, Johanna Wolf^2^, Kazumasa Saigo^3^, Shigekazu Kitamura^4^, Kumiko Ishizaki^1^, Junichi Miyahara^1^, Shoji Kikui^1^, Hiroo Yoshikawa^2^, Takao Takeshima^1^

#### ^1^Headache center, Tominaga hospital, Osaka, Japan; ^2^Division of Neurology, Department of Internal Medicine, Hyogo College of Medicine, Hyogo, Japan; ^3^Department of Life Science, Faculty of Science and Engineering, Kindai University, Osaka, Japan; ^4^Department of Neurology, Konan Hospital, Hyogo, Japan

##### **Correspondence:** Daisuke DANNO(daisukedanno@yahoo.co.jp)

*We certify that there is no actual and potential conflict of interest in relation to this study*

**Background:** Hemiplegic migraine (HM) is a rare headache disorder, and few data on its clinical symptoms are available in Japan.

**Method:** The clinical symptoms of 11 HM consecutive patients who visited the tertiary headache center in Japan were analyzed retrospectively.

**Results:** Of the 11 HM cases, 10 were sporadic HM (SHM), and 1 was familial HM (FHM). The patients were 13 to 67 years of age (average 36.2 years), including 7 males and 4 females. Headache was bilateral in 4 cases, unilateral side variable in 4 cases, and strictly unilateral in 2 cases. One case had no headache. The maximum headache intensity was NRS 9.7/10 on average. The longest headache duration was 39.6 h on average, with a maximum of 5 days. The four cases had allodynia. Aura symptoms preceded headache in two cases, aura and headache at the same time in two cases, and headache before aura in six cases. The motor symptoms were unilateral in nine cases and bilateral in two cases. Out of six cases of unilateral headache, ipsilateral headache was noted in four cases and contralateral headache in two cases. The progress of paralysis was the upper limbs followed by the lower limbs in four cases, at the same time in two cases, and the lower limbs followed by the upper limbs in two cases. The duration of paralysis was from 5 minutes to 2 months, with a median of 150 minutes, and in 2 cases, the paralysis was persistent. Sensory symptoms appeared in all cases. Speech symptoms were confirmed in eight cases and visual symptoms in seven cases. Brainstem symptoms were observed in eight cases, two of which showed a decreased level of consciousness. The order of occurrence of each aura was two cases along the order of visual sense, sensation, language, exercise, brain stem, but most of them were not constant. Genetic testing was performed in 10 cases, and SCN 1 A and ATP 1 A 2 were detected in 1 SHM case each.

**Discussion:** Many SHM cases are encountered at the tertiary headache center, some of which have persistent motor symptoms. The order of aura symptoms is diverse, with headache often preceding, and paralysis may be bilateral and ipsilateral. Given that some cases do not meet the diagnostic criteria, genetic testing is useful for confirming the diagnosis.

## A29 Functional connectivity changes within the salience network in patients with chronic migraine

### Francesca Pistoia^1,2^, Alessandra Splendiani^1,2^, Mario Quarantelli^1,2^, Antonio Carolei^1,2^, Paolo Cerrone^1,2^, Ilaria Frattale^1,2^, Simona Sacco^1,2^

#### ^1^Department of Biotechnological and Applied Clinical Sciences, University of L'Aquila, L'Aquila, Italy; ^2^Biostructure and Bioimaging Institute, National Research Council, Naples, Italy

##### **Correspondence:** Francesca Pistoia(francesca.pistoia@univaq.it)

Background: Migraine is a common neurological disorder whose pathogenesis is still unclear, especially when it occurs in its chronic form. The aim of the present study is to investigate functional connectivity within the salience network (SN) and the default mode network (DMN) in episodic migraine (EM) and chronic migraine (CM), in order to identify any functional brain connectivity changes associated with chronicization.

Methods: Patients consecutively referring to the Regional Headache Centre of L’Aquila with a diagnosis of migraine were screened for the inclusion in the study. The diagnosis of EM or CM was made according to the criteria of the International Classification of Headache Disorders (ICHD-III beta version). Twenty-four women with EM (mean age 45.5±8.7) and 24 women with CM (mean age 45.8±9.0) were included. Patients underwent a neuroradiological assessment through a 3 Tesla Magnetic resonance Imaging (MRI) scanner (Discovery MR750w). Resting-State fMRI data were analysed by means of a seed-based approach, using four and six different seeds, sampling the main hubs of the DMN and SN respectively.

Results: Alterations in functional connectivity were found in patients with CM as compared to patients with EM in the SN. Specifically, alterations involved the salience network between the left anterior insular cortex and the midcingulate cortex (p=0.0001) and the right supramarginal gyrus (p=0.0005). Further alterations were found in the connectivity between the right supramarginal gyrus and the left frontal operculum (p=0.0005).

Discussion: Our findings suggest that the SN, which is involved in the integration of sensory, emotional and cognitive information, may be dysregulated in patients with CM, especially in its crucial hubs represented by the anterior insula and the cingulate cortex. Dysfunctional connection among cortical hubs deserves further investigation in order to better understand migraine pathogenesis and to develop effective treatment strategies.

**References**

1. Schwedt TJ, Chiang CC, Chong CD, Dodick DW. Functional MRI of migraine. Lancet Neurol 2015;14:81-91.

2. Maleki N, Gollub RL. What Have We Learned From Brain Functional Connectivity Studies in Migraine Headache? Headache 2016;56:453-61.

3. Pistoia F, Sacco S, Carolei A. Behavioral therapy for chronic migraine. Curr Pain Headache Rep 2013;17:304.

## A30 Reduction in monthly migraine days (MMDs) with fremanezumab and erenumab among patients with chronic migraine (CM) with 2 to 4 prior treatment failures: A Network Meta-Analysis

### Rajeev Ayyagari^1^, Fan Mu^1^, Stephen Thompson^2^ , Akanksha Dua^1^, Ronghua Yang^2^, Yao Wang^1^, Joshua Cohen^2^, Sanjay Gandhi^2^

#### ^1^Analysis Group, Inc., Boston, MA; ^2^Teva Pharmaceuticals

##### **Correspondence:** Joshua Cohen(joshua.cohen05@tevapharm.com)

**Background**: We present a network meta-analysis (NMA) of the relative efficacy of CGRP monoclonal antibodies fremanezumab and erenumab in reducing monthly migraine days (MMDs) in chronic migraine (CM) patients with 2-4 prior treatment failures.

**Methods**: Randomized clinical trials of fremanezumab and erenumab (2007-present) were identified via a systematic literature review. Trials included adults with CM (≥15 headache days/month) who had 2-4 prior treatment failures. Reductions in MMDs at weeks 1-4 and weeks 1-12 were compared using pairwise treatment differences and 95% credible intervals (CrIs) obtained from Bayesian NMAs.

**Results**: Two trials evaluated reduction in MMDs for CM patients at weeks 1-4 and 1-12. Compared to placebo, MMD reduction was greater for fremanezumab monthly (675/225/225 mg) (weeks 1-4: 3.39 [95% CrI: 2.11-4.67]; weeks 1-12: 3.69 [2.43-4.96]), fremanezumab quarterly (675 mg/placebo/placebo) (weeks 1-4: 3.49 days [2.21-4.78]; weeks 1-12: 3.09 [1.84-4.35]), erenumab 140 mg (monthly) (weeks 1-4: 2.90 [1.40-4.43]; weeks 1-12: 3.90 [3.02-4.78]), and erenumab 70 mg (monthly) (weeks 1-4: 2.20 [0.68-3.73]; weeks 1-12: 2.50 [1.62-3.38]). Compared to erenumab 70 mg, MMD reduction was numerically greater for fremanezumab monthly (weeks 1-4: 1.19 [-0.81-3.19]; weeks 1-12: 1.20 [-0.36-2.74]) and fremanezumab quarterly (weeks 1-4: 1.29 [-0.70-3.30]; weeks 1-12: 0.59 [-0.94-2.15]). Compared to erenumab 140 mg, fremanezumab monthly and quarterly showed numerically greater reduction at weeks 1-4 (0.49 [-1.52-2.47] and 0.59 [-1.41-2.59]) and numerically less reduction at weeks 1-12 (-0.21 [-1.75-1.33] and -0.81 [-2.35-0.73]).

**Discussion**: In CM patients with 2-4 prior treatment failures, fremanezumab and erenumab had significantly greater reductions in MMDs than placebo, while there were no significant differences between fremanezumab and erenumab in reduction in MMDs over weeks 1-4 and weeks 1-12.

**Disclosures:** JC, SG, ST, and RY are employees of Teva Pharmaceuticals. RA, AD, FM, and YW are employees of Analysis Group Inc. which received consulting fees from Teva Pharmaceuticals.

## A31 Reduction in monthly migraine days (MMDs) with fremanezumab and erenumab among patients with episodic migraine (EM) with 2-4 prior treatment failures: A Network Meta-Analysis

### Fan Mu^1^, Oscar Patterson-Lomba^1^, Stephen Thompson^2^, Sanjay Gandhi^2^, Tahera Doctor^1^, Joshua Cohen^2^, Akanksha Dua^1^, Ronghua Yang^2^

#### ^1^Analysis Group, Inc., Boston, MA; ^2^Teva Pharmaceuticals;

##### **Correspondence:** Joshua Cohen(joshua.cohen05@tevapharm.com)

**Background**: We present a network meta-analysis (NMA) of fremanezumab and erenumab for reduction in monthly migraine days (MMDs) in patients with episodic migraine (EM) who failed 2-4 prior treatments.

**Methods**: RCTs of fremanezumab and erenumab were identified via a systematic literature review using MEDLINE, Embase, Cochrane Library, and Health Technology Assessment documents. All trials were published post 2007, and included adults with EM (<15 headache days per month) who had failed 2-4 prior preventive migraine treatments due to efficacy, safety, or tolerability. Reductions in MMDs during weeks 1-4 and weeks 1-12 were compared using pairwise treatment differences and 95% credible intervals (CrIs) obtained from Bayesian NMAs.

**Results**: Two trials were included for reduction in MMDs at weeks 1-4 and 1-12 among EM patients.

Over both durations, compared to placebo, MMD reduction was significantly greater for fremanezumab quarterly (675 mg/placebo/placebo) (weeks 1-4: 3.50 days [95% CrI: 2.24-4.77]; weeks 1-12: 3.10 [1.89-4.31]), fremanezumab monthly (225/225/225) (weeks 1-4: 3.50 [2.22-4.77]; weeks 1-12: 3.20 [1.97-4.41]), and erenumab 140 mg (monthly) (weeks 1-4: 1.70 [0.71-2.69]; weeks 1-12: 1.83 [1.21-2.45]). Reduction in MMDs at weeks 1-4 was significantly greater for fremanezumab quarterly vs. erenumab

140 mg (1.81 [0.19-3.43]) and for fremanezumab monthly vs. erenumab 140 mg (1.80 [0.19-3.41]). At weeks 1-12, fremanezumab monthly and quarterly had greater MMD reduction vs. erenumab 140 mg (1.37 [-0.01-2.73]) and 1.27 [-0.09-2.64] respectively).

**Discussion**: Among patients with EM with 2-4 prior treatment failures, fremanezumab and erenumab both had significantly greater reduction in MMDs than placebo, while fremanezumab had significantly greater reduction in MMDs than erenumab at weeks 1-4; for weeks 1-12, changes in MMDs were numerically larger than erenumab but did not reach statistical significance.

**Disclosures:** SG, JC, ST, and RY are employees of Teva Pharmaceuticals. AD, TD, FM, and OPL are employees of Analysis Group Inc., which received consulting fees from Teva Pharmaceuticals.

## A32 Comparison of responder rates between fremanezumab, erenumab and onabotulinumtoxinA among patients with migraine with ≥3 prior treatment failures: A Network Meta-Analysis

### Oscar Patterson-Lomba^1^, Rajeev Ayyagari^1^, Stephen Thompson^2^, Sanjay Gandhi^2^, Stephanie Bousleiman^1^, Ronghua Yang^2^, Yao Wang^1^, Joshua Cohen^2^

#### ^1^Analysis Group, Inc., Boston, MA; ^2^Teva Pharmaceuticals

##### **Correspondence:** Joshua Cohen(joshua.cohen05@tevapharm.com)

**Background**: We present a network meta-analysis (NMA) of responder rates (RR) of CGRP monoclonal antibodies and other common treatments in migraine patients with ≥3 prior treatment failures.

**Methods**: A systematic literature review of MEDLINE, Embase, Cochrane Library, and Health Technology Assessment documents was conducted to identify randomized clinical trials of fremanezumab, erenumab, and onabotulinumtoxinA (onaA) (2007-present). Adults with episodic (EM) or chronic migraine (CM) and ≥3 prior treatment failures were included. 30% RR in CM and 50% RR in EM were compared at the end of treatment period (weeks 9-12 or 21-24) between treatments using pairwise odds ratios (ORs) and 95% credible intervals (CrIs) from Bayesian NMAs.

**Results**: In CM patients, two trials evaluated 30% RR. Compared to placebo, improvements in 30% RR were significant for fremanezumab monthly 675/225/225 mg (4.03 [95% CrI: 2.06-8.15]), fremanezumab quarterly 675 mg/placebo/placebo (3.99 [2.04-8.00]), erenumab monthly 140 mg (3.05 [1.60-5.97]), and erenumab monthly 70 mg (2.47 [1.27-4.83]). The median ORs for 30% RR for fremanezumab monthly vs. erenumab 70 mg and 140 mg were 1.63 (0.63-4.34) and 1.32 (0.51-3.49), respectively. Fremanezumab quarterly showed similar results. In EM, two trials evaluated 50% RR. Compared to placebo, 50% RR were significant greater for fremanezumab 225/225/225 mg monthly (9.16 [2.95-36.02]), fremanezumab quarterly (11.52 [3.57-45.85]) and erenumab 140 mg (2.92 [1.20-7.81]). The median ORs for 50% RR for fremanezumab monthly and fremanezumab quarterly vs. erenumab 140 mg were 3.16 (0.70-15.62) and 3.98 (0.86-19.77), respectively.

**Conclusions**: Fremanezumab and erenumab had significantly higher 30% RR in CM and 50% RR in EM than placebo, and there was no significant difference in 30% RR in CM and 50% RR in EM between fremanezumab and erenumab.

**Disclosures:** JC, SG, ST, and RY are employees of Teva. RA, SB, OPL, and YW are employees of Analysis Group, Inc. which received consulting fees from Teva.

## A33 The effect of consulting a specially trained headache nurse on the quality of life of headache patients: controlled prospective interventional study

### Kristi Tamela^1,2^, Mark Braschinsky^1,2^

#### ^1^Neurology Clinic, Tartu University Hospital, Tartu, Estonia; ^2^Estonian Headache Society, Tartu, Estonia

##### **Correspondence:** Mark Braschinsky(mark.braschinsky@kliinikum.ee)

**Background.** Chronic headaches are difficult to treat, better management strategies and more efficient preventative care are needed. The goal of this study was to describe how consulting a specially trained headache nurse affects the quality of life (QoL) of headache patients.

**Methods.** Data was prospectively collected over the course of 2 years. The trial included patients of both genders (ages 18–65 years) with a diagnosis of a primary headache and no other significant comorbidity which could affect the QoL of the individual whom a neurologist had referred to a headache nurse consultation. Participants were randomly assigned to three groups. The first group received three consultations during the six month period: at the start of the trial and after three and six months. The second group was evaluated three times but the consultations took place twice: three and six months after the initial appointment. The control group was evaluated twice – first during the initial appointment and the second six months later; they received one consultation session at the end of the trial. Two tests were used for evaluation: Headache Impact Test (HIT-6) and Headache Under-Response to Treatment (HURT) survey.

The study was approved by the Research Ethics Committee of the University of Tartu.

**Results.** The trial included 80 patients; 64 of them attended all study appointments. The results of the study indicated that the QoL of patients who consulted a nurse improved remarkably compared to the control group. HIT-6 and HURT scores indicated substantial changes (p<0.01) in the mean scores of the first and second group. In the first group, the mean score of HIT-6 decreased from 63.3 to 56 at the time of the last appointment and the mean HURT score fell from 13.5 to 6.9. The second group scores dropped from 63 to 57 and from 13.5 to 7.1 respectively. The control group's mean scores of HIT-6 and HURT changed insignificantly.

**Discussion.** Consulting a headache nurse improves patients' QoL. The effect becomes apparent over a longer period of time. The results do not allow to conclude that a first consultation immediately after a doctor's appointment would be better for the QoL outcomes compared to a first consultation that took place three months after the initial appointment.

## A34 Botulinum toxin type-A treatment in chronic cluster headache: A case series of 19 patients

### Munoz-Delgado, L; Perez-Esteban, R; Jimenez-Hernandez, MD; ^1^Gonzalez-Oria, MC

#### Universitary Hospital Virgen del Rocio, Seville, Spain

##### **Correspondence:** Munoz-Delgado, L(lmunozdelgado12@gmail.com)

Background:Chronic cluster headache(CCH) pathophysiology involves an increase of calcitonin gene-related peptide(CGRP) plasma levels during attacks,on analogy to what happens in migraine. Effectiveness of botulinum toxin type-A(BTX-A) has been reported in chronic migraine by probably inhibiting the release of CGRP in trigeminal terminals. Similarities between both pathologies are the rationale basis to investigate the use of BTX-A for CCH therapy.

Methods: Descriptive retrospective single-center study with a prospectively collected database, including CCH patients treated with BTX-A according to the PREEMPT study protocol or modified unilateral PREEMPT protocol,repeated every three to four months. Simultaneous preventive medication was initially continued,being three patients carriers of occipital neurostimulators. Primary endpoints are effectiveness(measured as at least a 50% reduction in attack frequency or pain intensity compared to baseline) and reduction in disability(measured by HIT6 and HADS scores).Data obtained from diaries and medical registries.

Results: 19 patients were included,15 males,age range 29-76 years(mean 46.94(+/-11.63SD) and disease duration of 10.28 years(+/-9.42SD). Mean number of BTX-A dosis injected were 3(range 1-6) in an average follow-up time of 11.84(+6.74SD)months. Effectiveness in attack frequency and pain intensity was observed in 47.37% and 52.63% patients respectively. Improvement in disability scores was observed in 7 out of 11 patients. 26.32% patients achieved a reduction in the number of preventive medication.No adverse events were recorded.

Discussion: Our data show promising results regarding BTX-A treatment in CCH, not only in reducing the frequency or intensity of attacks, but also in improving quality of life. We highlight the sample size of our population.A more extensive follow-up time and experience are needed to establish which protocol may have better outcomes and whether patient characteristics predict response to BTX-A.

## A35 Intradermal administration of botulinum toxin type A (BTX-A) in patients with trigeminal neuralgia

### Perez-Esteban, R; Baena-Palomino, P; Lamas-Perez R.; Munoz-Delgado, L; Jimenez-Hernandez, MD, Gonzalez-Oria, MC

#### Universitary Hospital Virgen del Rocio (Seville, Spain)

##### **Correspondence:** Munoz-Delgado, L(lmunozdelgado12@gmail.com)

**Background:** Botulinum toxin type A(BTX-A) is one of the most potent neurotoxins, has been shown to be a choice of treatment for trigeminal neuralgia (TN), and may be an efficient, safe and novel strategy.

**Methods:** Prospective descriptive study. We included 16 patients with TN without response to 2 oral preventive treatments (OPT).The BTX-A administration protocol was: branches V2-V3, 25 IU/0.5 ml, distributed in 10 points for each branch and 3 contralateral points. Branche V1, according to the bilateral PREEMPT protocol, administered every 3 months.

The main objective was to assess the effectiveness:50% reduction in the intensity according to the visual analogic scale (EVAS) and/or the 50% reduction in the frequency of the crisis.

The secondary objectives were the tolerance and safety, the reduction OPT and disability scales (HitT-6 and the scale of anxiety and depression (ADH)).

**Results:** 16 patients were included,7 males(43.8%),age range 40-86 years(mean 62.4 ±14.6SD). Mean follow-up time:73 months (IQR of 19-363 months); mean number of Botox doses was 3 (range 1-7,IQR 2). Had been treated with a median of 4 OPT (range 2-5). The proportion of intensity responders was 81.25% and 62,7% of frequency responders.4 patients (25%) discontinued the study. Median number of preventive drugs was reduced to 3 (range 0-4). In the disability scales, the average reduction was: 8.21(SD ± 12.2) for HIT-6 scale, 3.57(SD ± 5.0)for ADH-anxiety and 2.64 (SD ± 4.2) points for ADH-depression .

**Discussion:** The administration of BTX-A for trigeminal neuralgia is effective, and associated with a reduction in number of preventive drug and in disability scales (Hit-6 and ADH).Adverse effects are frequent(43.8%)but not incapacitating.

## A36 Prophylactic effect of ultramicronized N-Palmitoyl Ethanol Amide (PEA) on pediatric migraine

### Laura Papetti^1*^, Giorgia Sforza^1,2*^, Giulia Tullo^3^, Samuela Tarantino^1^, Romina Moavero^1,2^, Fabiana Ursitti^1^, Michela Ada Noris Ferilli ^1^, Massimiliano Valeriani^1,4^.

#### ^1^Headache Center, Child Neurology Unit, Bambino Gesu’ Children’s Hospital, Rome, Italy; ^2^Child Neurology and Psychiatry Unit, Tor Vergata University of Rome, Italy; ^3^Pediatric Unit, Sant’Andrea Hospital, University Sapienza of Rome, II faculty; ^4^Center for Sensory-Motor Interaction, Aalborg University, Aalborg, Denmark Neurology Unit.

##### **Correspondence:** Massimiliano Valeriani(massimiliano.valeria@OPBGMAIL.onmicrosoft.com)

*These authors have equally contributed.

**Background:** Palmitoyl ethanolamide (PEA) is an amide of endogenous fatty acids widely distributed in different tissues, including nervous tissues. PEA is emerging as a new therapeutic approach in pain and inflammatory conditions and it has been evaluated in studies on various painful diseases. However, to date no studies have been conducted to evaluate the role of PEA in the management of migraine in pediatric patients.

*OBJECTIVE:* ***The aim of this open-label study was to evaluate the efficacy of ultramicronized PEA (um-PEA) in the prophylactic treatment of migraine.***

**Methods:** The study included 53 patients with mean age of 10.67 ± 3.1 (24.5% M and 75.5% F). All patients had a diagnosis of migraine without aura (ICHD 3 criteria) and received umPEA (600 mg/day orally) for three months. We compared the attack frequency (AF) and attack intensity at baseline and after three months. Patients were asked to classify the intensity of the attack with a value ranging from 1 to 3 where 1 means mild attack, 2 moderate and 3 severe attack.

**Results:** After 3 months of treatment with um-PEA, the headache frequency was reduced by >50 % per month in 56.6% patients. Three patients discontinued treatment too early (less than a month) and were not considered in the results. After three months of treatment the number of monthly attacks decreased significantly compared to the start of therapy (from 13.89 ± 7.6 SD to 6.43 ± 5.1 SD; p<0.05) The intensity of the attacks went from 1.70 ± 0.6 (pre-PEA) to 1.19 ± 0.5 (post-PEA).

**Discussion:** Our preliminary data show that um-PEA administered for three month reduces pain intensity and the number of attacks per month in in pediatric patients with migraine. Although the small number of patients and the lack of control group do not allow us to consider these initial results as definitely reliable, they encourage us to expand the sample.

## A37 A case of supraorbital neuralgia successfully treated with pregabalin

### Koutsokera, Pasqua Acquaviva

#### Department, "Thriassio" General Hospital, Elefsis, Greece

##### **Correspondence:** Maria Koutsokera(mkoutsokera@yahoo.gr)

Background: Supraorbital neuralgia is a rare disorder characterized by unilateral pain involving the supraorbital region meeting the following diagnostic criteria set by the International Headache Society Classification (ICHD-II): A) forehead pain in the territory supplied by the supraorbital nerve, without side shift; B) tenderness on either the supraorbital notch or traject of the nerve; and C) absolute, but transitory relief of symptoms upon supraorbital nerve blockade.

Case study: A 51-year-old male with a 5-year history of severe stabbing intermittent pain in the right frontal region referred to our department because of increasing frequency and intensity of the pain over the last few months. The symptoms usually emerged during exposure to cold wind while recently appeared even with light touch being quite disabling for the patient. There was no past history of trauma but he used to wear a tight-fitting motorcycle helmet. On palpation, there was a tender area over the exit of the right supraorbital nerve. Neurological assessment and brain imaging was normal. The patient was advised to change his helmet and to receive pregabalin to a total dose of 150mg. The patient improved significantly and after a 6-month-period, he gradually discontinued the medication without recurrence.

Discussion: Our patient fulfilled the criteria A and B set by ICHD-II for supraorbital neuralgia. He also exhibited allodynia and exteroceptive precipitating mechanisms usually seen in cases related to head trauma, although he did not reported any. Due to the rarity of the condition and its similarity of presentation as other headaches, it is important to correctly diagnose and treat the patient. Pregabalin should be considered as a possible treatment for supraorbital neuralgia other than supraorbital nerve block.

Written, informed consent for publication was obtained from the patient.

## A38 Cyclic vomiting syndrome and benign paroxysmal torticollis are associated with a high risk of developing primary headache: a longitudinal study

### Michela Ada Noris Ferilli^1,^ Romina Moavero^1,2^, Laura Papetti^1^, Maria Chiara Bernucci^1^, Caterina Cenci^1^, Giorgia Sforza^2^, Federico Vigevano^1^, Massimiliano Valeriani^1,3^

#### ^1^Headache Center, Bambino Gesù Children’s Hospital, IRCCS, Rome, Italy; ^2^Child Neurology and Psychiatry Unit, Tor Vergata University of Rome, Italy; ^3^Center for Sensory Motor Interaction Aalborg University, Aalborg, Denmark

##### **Correspondence:** Massimiliano Valeriani(massimiliano.valeria@OPBGMAIL.onmicrosoft.com)

**Abstract**

**Background.** Periodic migraine variants are a group of disorders affecting patients with migraine or with an increased risk of presenting it, and likely represents an early life expression of migraine. Cyclic vomiting syndrome (CVS) and benign paroxysmal torticollis (BPT) are well characterized and represent a frequent cause of request for specialistic consultations. Aim of this study is to longitudinally assess the rate of headache in patients presenting with CVS and BPT during infancy, and to define the main clinical features of the disorder. **Methods**. We administered a questionnaire to the parents of all our pediatric patients with previous diagnosis of CVS and/or BPT according to ICHD-3; questions were focused on the main clinical features of the disorder as well as the prognosis, with particular emphasis on the development of headache. **Results**. For the final analysis we considered 82 patients with CVS and 33 with BPT. Seventy-nine percent of patients with CVS presented with headache during the follow-up, with a mean age at onset of 6 years; 67% of patients with BPT suffered from headache during the follow-up, with a mean age at onset of 5 years. **Discussion**. CVS and BPT are associated with a very high risk of developing headache, mostly migraine, later in life. In both groups of patients, the vast majority presented with different periodic migraine variants at different ages, thus suggesting an age dependent evolution of migraine-like symptoms before the onset of clear migrainous headache.

## A39 Dysphasia and other higher cortical dysfunctions during the migraine aura - A systematic review of literature

### Igor Petrusic^1^, Michele Viana^2,3*^, Chiara Zecca,^2^, Jasna Zidverc-Trajkovic ^4,5^

#### ^1^Laboratory for advanced analysis of neuroimages, Faculty of Physical Chemistry, University of Belgrade, Serbia; ^2^Headache Center, Institute of the Neurocenter of Southern Switzerland (NSI), Regional Hospital Lugano, Lugano, Switzerland; ^3^Headache Group, Department of Basic and Clinical Neurosciences, King's College London, London, UK; ^4^Center for headaches, Neurology Clinic, Clinical Center of Serbia; ^5^Faculty of Medicine, University of Belgrade, Serbia

##### **Correspondence:** Michele Viana(michele.viana@ymail.com)

Conflict of interest: None

**Abstract**

**Background:** Visual and somatosensory disturbances are the most common symptoms of migraine aura (MA). Yet, patients can experience also other disturbances during their MA that can be defined higher cortical dysfunctions (HCDs). HCDs can belong to visual group (i.e. prosopagnosia), somatosensory group (i.e. astereognosis, dyspraxia, and neglect of own body parts), language group (i.e. Broca's dysphasia, Wernicke's dysphasia, and dysnomia) and memory group (e. g. difficulties to remember or recall events, recall names, and calculating and/or memorizing numbers).

**Methods:** We performed a systematic literature search in order to identify all the studies evaluating symptoms of HCDs during MA.

**Results:** We identified five studies including 697 patients. Overall, symptoms of HCDs occurred at a frequency of 10 to 65% of subjects, and the most frequently reported was dysphasia (range 10-53%). Visual HCDs occurred in 13-40% of the patients. Somatosensory HCDs were reported in 12-20% (manual dyspraxia being the most frequently reported), while memory disturbances were noted in 10-15% of the patients.

**Discussion:** MA can be associated with a wide range of neurological symptoms, among which symptoms of HCDs seem to be quite frequent according to our literature review. Despite this, HCDs in MA have been poorly investigated so far and, with the exception of dysphasia, are not included in ICHD-III among other MA disturbances. We believe such symptoms should be further investigated contributing to a better understanding of MA pathophysiology and to a proper stratification of patients in the research setting.

## A40 Poor sleep quality in tension-type headache: a population-based study

### Tae-Jin Song^1^; Kyung Min Kim^2^, Soo-Jin Cho^2^; Won-Joo Kim^3^; Kwang Ik Yang^4^; Chang-Ho Yun^5^; Min Kyung Chu^1,3^ +82-2-2228-1600

#### ^1^Department of Neurology, Ewha Womans University College of Medicine; ^2^Department of Neurology, Dongtan Sacred Heart Hospital, Hallym University College of Medicine, Hwaseong, Korea; ^3^Department of Neurology, Gangnam Severance Hospital, Yonsei University, College of Medicine, 50-1 Yonsei-ro Seodaemun-gu, Seoul 03772, Republic of Korea; ^4^Department of Neurology, Soonchunhyang University College of Medicine, Cheonan Hospital, Cheonan, Korea^4^; ^5^Department of Neurology, Bundang Clinical Neuroscience Institute, Seoul National University Bundang Hospital, Seongnam, Korea

##### **Correspondence:** Min Kyung Chu(chumk@yonsei.ac.kr)

**Conflict of Interest Statement**

Tae-Jin Song: None.

Soo-Jin Cho was a site investigator of a multicenter trial sponsored by Otsuka Korea, Eli Lilly and Company, Korea BMS, and Parexel Korea Co., Ltd.. Soo-Jin Cho also worked as an advisory member for Teva. Soo-Jin Cho received research support from Hallym University Research Fund 2016 and Academic award of Myung In Pharm. Co. Ltd. Soo-Jin Cho also received lecture honoraria from Yuyu Pharmaceutical Company and Allergan Korea.

Won-Joo Kim: None.

Kwang Ik Yang: None.

Chang-Ho Yun: None.

Min Kyung Chu was a site investigator for a multicenter trial sponsored by Otsuka Korea, Novartis International AG and Eli Lilly and Company. Min Kyung Chu worked an advisory member for Teva and received lecture honoraria from Allergan Korea and Yuyu Pharmaceutical Company in the past 24 months.

**Keywords:** depression, epidemiology, headache, migraine, Patient Health Questionnaire-9

**Funding**

This study was supported by a 2011 grant from the Korean Academy of Medical Sciences.

**Abbreviations**

KHSS, Korean Headache-Sleep Study; ICHD-II, The second edition of the International Classification of Headache Disorders; PHQ-9, Patient Health Questionnaire-9; DSM, Diagnostic and Statistical Manual of Mental Disorders; VAS, Visual Analogue Scale; HIT-6, Headache Impact Test-6; 5-HT, 5-Hydroxytryptamine; WHO, World Health Organization;

**Acknowledgments**

The authors would like to thank Gallup Korea for providing technical support for the Korean Headache-Sleep Study.

**Abstract**

Background: Tension-type headache (TTH) represents the most common type of headache among the general population. Although such headaches are usually mild in severity, some individuals with TTH experience severe symptoms and psychiatric comorbidities. Such patients may also experience sleep disturbances, which have been associated with headache exacerbation. However, information on the prevalence and clinical implication of poor sleep quality among individuals with TTH is scarce. Thus, the aim of this study was to assess the prevalence and clinical impact of poor sleep quality in individuals with TTH in a population-based setting.

**Methods:** We used data from the Korean Headache-Sleep Study, a nation-wide survey regarding headache and sleep for adults aged 19-69 years. Depression was defined as Patient Health Questionnaire-9 score ≥ 10.

**Results:** Of 2,695 participants who completed the survey, 570 (21.2%) had TTH and 715 (26.5%) had poor sleep quality. Among individuals with TTH, the prevalence of poor sleep quality among individuals with TTH participants with ≥15 headache frequency per month (50.0%) was significantly higher than TTH participants with 1-14 headache frequency (36.7%, p<0.001), TTH participants with <1 headache frequency per month (24.2%, p<0.001). For subjects with TTH, headache frequency per month (3.0±6.1 vs. 1.5±4.1, p=0.006), visual analogue scale score for headache intensity (5.0[3.0-6.0] vs. 4.0[3.0-5.0], median and interquartile range, p=0.003) and the Headache Impact Test-6 (45.8±6.9 vs. 43.0±6.1, p<0.001) were higher in participants with poor sleep quality than in those without poor sleep quality.

**Conclusions:** Our findings suggest that poor sleep is linked to an exacerbation of TTH. Therefore, the proper evaluation and management of sleep may lead to the better management of TTH. The prevalence of poor sleep quality between individuals with TTH and those with non-headache did not significantly differ (34.5% vs. 28.7%, p=0.224).

## A41 Headache and brain vascular malformations: pitfalls in the diagnostic

### Olena Tsurkalenko, Lyudmyla Dzyak

#### SI "Dnipropetrovsk medical akademy" Dnipro, Ukraine

##### **Correspondence:** Olena Tsurkalenko(alena.tsurkalenko@gmail.com)

Background: brain vascular malformations (BVM) are complex vascular lesions commonly associated with chronic headache, which could be the first manifestation of the disease. However, the specific characteristics of BVM-assotiated headache are still poorly described. TMethods: Between 2005 and 2018 years 397 consecutive patients with BVM were managed. We conducted a comprehensive clinical, neuropsychological and neuroimaging examination of this subjects. Headache was characterized according to ICHD-II criteria.

Results: In our study, 68% of women and 54% of men were found to have headache (42% had migraine-like headache without aura, 29% - migraine-like headache with aura, 25% - tension-type headache, 14% - trigeminal-like autonomic cephalgias). In 206 patients (52%) it was the first symptom of the disease. Clinical and radiological findings of BVM images showed that headache occurred significantly more frequently among larger malformations (with vs. without headache, 12.7 vs. 5.1 ml, p=0,002), with transdural arterial communication (84.6 vs. 29.7%, p = 0.001), occipital and midbrain BVM (69.5 vs. 29.1%, p < 0.001), older patients (43.1 vs. 36.6 years, p = 0.037). It should be marked that only 32% of headache patients with BVM completely meet ICH-III criteria for primary headaches, 68% patients - have specific headache features or additional symptoms, allowed to suspect their secondary character.

Discussion: Intractable, primary-like headaches, which dont completely meet ICH-III criteria, should increase suspicion for BVM and prompt neuroimaging. The pathogenesis of headache in BVM patients may involve several mechanisms and demonstrate general lesion of the brain vessels caused by disgenesis. Unfortunatelly, causality is far from clear and need future investigations.

## A42 Consistent reductions in migraine frequency with eptinezumab treatment in patients with migraine stratified by disease characteristics: subgroup analysis of PROMISE-1 and PROMISE-2

### George Chakhava^1^, Roger Cady^2^, Eric Kassel^2^, Joe Hirman^3^, Steve Snapinn^2^

#### ^1^Multiprofile Clinic Consilium Medulla, Georgian Association of Medical Specialties, Tbilisi, Republic of Georgia; ^2^Alder BioPharmaceuticals, Inc., Bothell, WA, United States; ^3^Pacific Northwest Statistical Consulting, Inc., Woodinville, WA, United States

##### **Correspondence:** Roger Cady(rcady@alderbio.com)

**Background:** In two phase 3 clinical trials of episodic migraine (EM) (PROMISE-1; NCT02559895) and chronic migraine (CM) (PROMISE-2; NCT02974153), eptinezumab met the primary efficacy endpoint. Treatment response cannot be assumed as homogenous across subgroups. This subanalysis reports prespecified subgroup analyses of the impact of disease characteristics on the primary endpoint in PROMISE-1 and PROMISE-2.

**Methods:** Eligible patients with EM or CM were randomized to repeat doses of eptinezumab 100mg, 300mg, or placebo (or 30mg in PROMISE-1), administered intravenously every 3 months for up to 4 (PROMISE-1) or 2 (PROMISE-2) infusions. The primary endpoint was the change from baseline in mean monthly migraine days (MMDs) over Months 1-3. Here, mean MMDs were analyzed in patient subgroups defined by disease characteristics including: duration of migraine (≤15/>15 years), baseline MMDs (≤9/>9 days, PROMISE-1; <17/≥17 days, PROMISE-2), baseline triptan use (<33%/≥33% of days/month, PROMISE-2), concomitant prophylactic medication use (yes/no, PROMISE-2).

**Results:** In all subgroups with ≥100 patients, the mean change from baseline in MMDs over Months 1-3 favored eptinezumab over placebo. PROMISE-1 difference from placebo in 100mg and 300mg, respectively: total, -0.7 (p=0.0001), -1.1 (p=0.0182); migraine duration ≤15y, -0.4, -1.5 vs >15y, -1.3, -0.8; baseline MMDs ≤9d, -1.1, -1.0 vs >9d, -0.3, -1.5. PROMISE-2: total, -2.0 (p<0.0001), -2.6 (p<0.0001); migraine duration ≤15y, -2.0, -2.3 vs >15y, -1.9, -2.8; baseline MMDs <17d, -2.6, -3.1 vs ≥17d, -1.2, -1.8; triptan use <33%, -2.1, -2.1 vs ≥33%, -1.8, -3.4; yes concomitant prophylactic medication, -1.4, -2.0 vs no concomitant, -2.4, -2.9.

**Discussion:** Subgroup analyses are important for determining if an overall treatment effect is consistent across the full trial population. Eptinezumab showed consistent reductions from baseline in mean MMDs vs placebo across clinically relevant disease characteristics in patients with migraine.

**Funding/Sponsor:** Funding and support provided by Alder BioPharmaceuticals, Inc., Bothell, WA, USA.

## A43 Content validity of the HIT-6 in migraine patients: results of a systematic literature review

### Carrie R. Houts^1^, RJ Wirth^1^, James S. McGinley^1^, Chad Gwaltney^2^, Roger Cady^3^

#### ^1^Vector Psychometric Group, LLC, Chapel Hill, NC, United States; ^2^Gwaltney Consulting, Westerly, RI, United States; ^3^Alder BioPharmaceuticals, Inc., Bothell, WA, United States

##### **Correspondence:** Roger Cady(rcady@alderbio.com)

**Background**: The short-form Headache Impact Test (HIT-6) is a patient-reported outcome measure that assesses the negative impact of headaches on normal activity. As it was not specifically developed using feedback from/tailored to patients with migraine, its use in this population has been criticized. The objective of this systematic literature review was to identify qualitative evidence of the content validity of the HIT-6 with respect to patients with migraine.

**Methods:** English-language reports of HIT-6 use were identified through systematic searches of PubMed and Google Scholar using *a priori* specified search terms. Independent reviewers identified eligible studies (those with qualitative research or psychometric results regarding HIT-6 content) and summarized evidence of content validity.

**Results:** Twelve publications were identified with supportive qualitative evidence for HIT-6 content validity, 8 specific to migraine (episodic and chronic) and 4 citing general headache patients. The majority of publications utilized either 1-on-1 patient interviews or patient-centered focus groups. There were 283 patients interviewed across studies: 214 patients with migraine, including 16 identified as patients with chronic migraine.

Overarching themes and specific information (e.g., patient quotes) supporting the relevance of content of each HIT-6 item to migraine patients’ lives were found. Across interviews, limitations in daily activities, needing to lie down during headaches, feeling tired, being irritated by headaches, and difficulty concentrating were identified as relevant effects of headaches on patients with migraine. Additionally, research indicated that patients understood the HIT-6 instructions, items, and response scales as intended by the instrument authors.

**Discussion:** This systematic literature review provides valuable support for the content validity of the HIT-6 for use in patients with migraine.

**Funding/Sponsor**

Financial Support: Funding and support provided by Alder BioPharmaceuticals, Inc., Bothell, WA, USA.

## A44 Establishing a responder definition for the HIT-6 items in a chronic migraine population

### Richard Lipton^1^, Carrie R. Houts^2^, Joe Hirman^3^, RJ Wirth^2^, James S. McGinley^2^, Roger Cady^4^

#### ^1^Albert Einstein College of Medicine, Bronx, NY, United States; ^2^Vector Psychometric Group, LLC, Chapel Hill, NC, United States; ^3^Pacific Northwest Statistical Consulting, Inc., Woodinville, WA, United States; ^4^Alder BioPharmaceuticals, Inc., Bothell, WA, United States

##### **Correspondence:** Roger Cady(rcady@alderbio.com)

**Background:** The amount of within individual change on the short-form Headache Impact Test (HIT-6) needed over time to be interpreted as meaningful (responder) has been defined in several headache populations, but important changes at the item level have not been examined. The objective of this analysis was to identify responder definitions for the individual HIT-6 items in patients with chronic migraine (CM).

**Methods:** Responder definitions for the HIT-6 items were analyzed using the PROMISE-2 (NCT02974153) study population of patients with CM (N=1024 available at Week 12). HIT-6 responses were scored using integers (from 1=never to 5=always). Multiple anchor-based methods were used to establish a responder threshold: patient global impression of change, ≥75% reduction in migraine frequency, and EuroQOL 5-dimensions 5-level (EQ-5D-5L) visual analog scale. Resulting candidate responder values were plotted against the cumulative distribution function of change values (baseline to week 12) and used to triangulate to empirically supported responder definitions for the HIT-6 items in patients with CM.

**Results:** Responder definition thresholds for individual HIT-6 items were found to be an improvement of 1 category on items 1 (pain severity), 2 (limits daily activities), and 3 (wish to lie down) and improvement of 2 categories on items 4 (too tired to work), 5 (felt fed up or irritated), and 6 (limits concentration). For all 3 anchor-based analyses across each of the 6 items, the improved group exhibited greater mean improvement in HIT-6 item scores than the reference (no change/worse) group.

**Discussion:** For patients with CM, improvements of 1 category on items 1–3 or 2 categories on items 4–6 of the HIT-6 appear most appropriate for identifying CM patients who have experienced meaningful change at the item level. These results can be used to facilitate the interpretation of HIT-6 findings in clinical trials and by clinicians.

**Funding/Sponsor**

Financial Support: Funding and support provided by Alder BioPharmaceuticals, Inc., Bothell, WA, USA.

## A45 Consistent reductions in migraine frequency with eptinezumab treatment in patients with migraine stratified by intrinsic factors: subgroup analyses of PROMISE-1 and PROMISE-2

### Marina Janelidze^1^, Gvantsa Giorgadze^2^, Roger Cady^3^, Steve Snapinn^3^, Joe Hirman^4^, Eric Kassel^3^

#### ^1^Department of Neurology, Tbilisi State Medical University, Tbilisi, Republic of Georgia; ^2^Department of Neurology, LTD Aversi Clinic, Tbilisi, Republic of Georgia; ^3^Alder BioPharmaceuticals, Inc., Bothell, WA, United States; ^4^Pacific Northwest Statistical Consulting, Inc., Woodinville, WA, United States

##### **Correspondence:** Roger Cady(e-mail: rcady@alderbio.com)

**Background:** In two phase 3 clinical trials of episodic migraine (EM) (PROMISE-1; NCT02559895) and chronic migraine (CM) (PROMISE-2; NCT02974153), eptinezumab met the primary efficacy endpoint. Treatment response cannot be assumed as homogenous across subgroups. This subanalysis reports prespecified subgroup analyses of the impact of intrinsic factors on the primary endpoint in PROMISE-1 and PROMISE-2.

**Methods:** Eligible patients with EM (PROMISE-1) or CM (PROMISE-2) were randomized to repeat doses of eptinezumab 100mg, 300mg, or placebo (or 30mg in PROMISE-1), administered intravenously every 3 months for up to 4 (PROMISE-1) or 2 (PROMISE-2) infusions. The primary endpoint in both studies was the change from baseline in mean monthly migraine days (MMDs) over Months 1-3. Here, mean MMDs were analyzed in patient subgroups defined by intrinsic factors including: age (≤35/>35 years), sex (male/female), race (white/black).

**Results:** In all subgroups with ≥50 patients, the mean change from baseline in MMDs over Months 1-3 favored eptinezumab over placebo. PROMISE-1 difference from placebo: total population, -0.7 (100mg, p=0.0001), -1.1 (300mg, p=0.0182); age ≤35y, -1.2 (100mg), -1.0 (300mg) vs >35y, -0.7 (100mg), -1.4 (300mg); male, -0.5 (100mg), -0.8 (300mg) vs female, -0.9 (100mg), -1.3 (300mg); white, -1.0 (100mg), -1.4 (300mg) vs black, 0.2 (100mg), -0.2 (300mg). PROMISE-2: total population, -2.0 (100mg, p<0.0001), -2.6 (300mg, p<0.0001); age ≤35y, -2.6 (100mg), -3.0 (300mg) vs >35y, -1.6 (100mg), -2.3 (300mg); male, -2.4 (100mg), -2.6 (300mg) vs female, -1.9 (100mg), -2.5 (300mg); white, -2.2 (100mg), -2.8 (300mg) vs black, -0.5 (100mg), 0 (300mg).

**Discussion:** Subgroup analyses are important for determining if an overall treatment effect is consistent across the full trial population. Eptinezumab treatment showed consistent reductions from baseline in mean MMDs when compared with placebo across clinically important intrinsic subgroups.

**Funding/Sponsor:** Funding and support provided by Alder BioPharmaceuticals, Inc., Bothell, WA, USA.

## A46 Is pediatric medication overuse headache really due to medication overuse?

### Romina Moavero, Maddalena Stornelli, Laura Papetti, Barbara Battan, Samuela Tarantino, Federico Vigevano, Massimiliano Valeriani

#### Headache Center, Bambino Gesù Children’s Hospital, IRCCS, Rome, Italy

##### **Correspondence:** Massimiliano Valeriani(massimiliano.valeria@OPBGMAIL.onmicrosoft.com)

**Background**. Medication Overuse Headache (MOH) is a headache occurring on ≥15 days/month in patients with pre-existing primary headache and developing as a consequence of regular overuse of symptomatic medication. Aim of this study was to analyze the clinical features of pediatric MOH, with particular emphasis on the applicability of ICHD-3 criteria. **Methods**. We retrospectively analyzed clinical data of pediatric patients with MOH; the clinical diagnosis was verified according to ICHD-3 criteria. Although no more included in the diagnostic criteria, we analyzed how many patients presented a clinical benefit after discontinuation of overused medication. **Results**. We identified 42 subjects diagnosed with MOH (31 F, 11 M), aged 8-17 years (mean 13.4 years). They all presented with chronic migraine, 9% fulfilled a diagnosis of migraine with aura. Photo- and photophobia were present in 81% of patients, nausea/vomiting in 30%, dizziness in 18%. ICHD-3 criterion A was fulfilled by 40/42 patients (95%), criterion B by 35/42 (83%), and criterion C by 40/42 (95%). Nineteen patients (45%) did not present an improvement of headache after medication overuse cessation. **Discussion**. The old criteria required a development or marked worsening of the headache during medication overuse, and a resolution within 2 months after medication withdrawal. Both these criteria disappeared in ICHD-3. Our data show that, without the necessity of demonstrating a clear and direct correlation with abuse and discontinuation of symptomatic medications, a definite diagnosis can be achieved in a high rate of patients with a clinical suspicion of MOH. Nearly half of patients with MOH didn’t improve after medication overuse cessation, thus raising the doubt of a true causal relationship between medication overuse and chronic headache. **Conclusion**. A high rate of patients with a definite diagnosis of MOH according to new criteria continued to present a high frequency headache despite the withdrawal of overuse.

## A47 Role of maternal stress and alexithymia in children’s migraine severity and psychological profile

### Samuela Tarantino^1^, Alessandra di Stefano^1^, Valeria Messina^1^, Laura Papetti^2^, Michela Ferilli^2^, Giorgia Sforza^3^, Fabiana Ursitti^2^, Romina Moavero^2^, Federico Vigevano^2^, Simonetta Gentile^1^ and Massimiliano Valeriani^2,4^

#### ^1^Unit of Clinical Psychology, ^2^Headache Center, Division of Neurology, Ospedale Pediatrico Bambino Gesù, IRCCS, Piazza Sant’Onofrio 4, Rome, Italy; ^3^Child Neurology and Psychiatry Unit, Tor Vergata University of Rome; ^4^Center for Sensory-Motor Interaction, Aalborg University, Aalborg, Denmark

##### **Correspondence:** Massimiliano Valeriani(samuela.tarantino@opbg.net)

**Background**. Recent studies showed that patients’ attachment style and maternal alexithymia traits may impact on psychological profile and pain expression in children/adolescents suffering from migraine. So far, very few studies explored the relationship between maternal stress, children’s psychological profile and migraine severity. Aims of our study were to explore the role of maternal parenting stress and alexithymia on: 1) children’s headache severity (frequency); 2) maternal perception of children’s psychological conditions and 3) children’s psychological profile.

**Methods**. We studied 51 migraineurs (mean age 11.6 ± 2.1 years; 22 M and 29 F). Patients were divided in two groups according to the headache attacks frequency (high and low). Maternal stress and alexithymia levels were evaluated by PSI-SF and TAS-20 questionnaires. We used SAFA “Anxiety” and “Depression” scales to explore children's psychological profile. To evaluate maternal perception of children’s psychological conditions CBCL 6/18 was employed. **Results**. We found a correlation between maternal stress and CBCL Internalizing (p= 0.00), Externalizing (p= 0.00) and Total scales (p= 0.00). A positive correlation has been identified between mothers’ PSI Total score and SAFA-D Total score (p= 0.03). In particular, a positive correlation was found between “Parental distress” and children’s SAFA-D “Feeling of guilt” subscales (p= 0.04). Maternal stress and alexithymia did not show significant differences among the two migraine frequency groups (p >0.05). However, in high frequency group, PSI Total score showed a positive correlation with Internalizing scale (p= 0.00). No relationships were found between TAS-20, CBCL, SAFA and migraine frequency. **Conclusions**. Maternal stress has no relationship with children’s migraine frequency. However, it shows a relationship with maternal perception of children’s psychological profile and patients’ depressive symptoms, which in turn may impact on migraine severity.

## A48 Is it useful to determine CGRP, VIP, and PACAP in migraine? A cross-sectional study in chronic migraine patients

### Sara Pérez-Pereda^1^, María Toriello-Suárez^1^, Gonzalo Ocejo-Vinyals^2^, Sandra Guiral-Foz^2^, Jesús Castillo-Obeso^3^, Silvia Montes-Gómez^3^, Rosa Maria Martínez-Nieto^3^, Vicente González-Quintanilla^1^, Agustín Oterino-Durán^1^

#### ^1^Neurology Service University Hospital Marqués de Valdecilla and IDIVAL. Cantabria. Spain; ^2^Inmunology Service University Hospital Marqués de Valdecilla. Cantabria. Spain; ^3^Primary Care Camargo Costa Health Center. Cantabria. Spain

##### **Correspondence:** Sara Pérez-Pereda(sperez@idival.org)

**Background:** VIP, CRGP, and PACAP have important roles in migraine pathogenesis but their diagnostic value is controversial. Objective: To determine and compare these neuropeptides serum levels in a cohort of chronic migraine (CM), episodic migraine (EM), and healthy controls (HC).

**Methods:** Peripheral blood samples from 304 age and sex matched subjects (28 males and 276 females: 108 CM, 98 EM and 98 HC) were drawn, centrifuged, and stored at -80º C. ELISA-based assays were performed using BlueGene Biotech Co kit for PACAP and Cloud-Clone Corp kit for CGRP and VIP. Standardized values were compared using t-test for univariate analysis. A multinomial regression analysis was performed to identify predictors of clinical diagnosis. Pearson’s “r” statistic was used for bivariate correlation analysis and Chi2 for categorical variables. Area under the curve (AUC) in ROC curves were used to assess their diagnostic value.

**Results:** VIP, PACAP and CGRP were increased in CM vs EM and HC (p<0,001), but not in EM vs HC. Age inversely correlated with PACAP and CGRP but not with VIP. Significant correlation occurred between VIP and PACAP (r=0.347), VIP and CGRP (r=0.526), and PACAP and CGRP (r=0.504). Only VIP (B=0.009) and PACAP (B= 003) predicted the clinical diagnosis for CM, but not EM. GCRP did not predict CM nor EM. Only 60.2% of CM, 68.8% of EM, and 15.5% of HC were correctly predicted. AUC were significant for VIP and PACAP (0.703±0.33, 0.739±0.32, respectively). Both had low sensibility for CM (0.53 and 0.50 respectively), and better specificity (0.83 and 0.939 respectively).

**Conclusion:** We found that VIP, CGRP and PACAP were significantly increased in CM. In the present study CGRP value as a CM biomarker was relatively low. Standardized techniques for analyzing these neuropeptides are strikingly needed. Funded by ISCIII-FISS PI15/01285 and IDIVAL.

## A49 Protective role of fruit and vegetable against pediatric migraine headache: A case- control study

### Soodeh Razeghi Jahromi^1,2^, Shadi Ariyanfar^1,2^, Nasim Rezaeimanesh^3,1^, Mansoureh Togha^2^ Zeinab Ghorbani^2,4^, Ebrahim Khadem^5^

#### ^1^Department of Clinical Nutrition and Dietetics, Faculty of Nutrition and Food Technology, National Nutrition and Food Technology Research Institute, Shahid Beheshti University of Medical Sciences, Tehran, Iran; ^2^Headache Department, Iranian Center of Neurological Research, Neuroscience Institute, Tehran University of Medical Sciences, Tehran, Iran; ^3^Multiple sclerosis Research Center, Neuroscience Institute, Tehran University of Medical Sciences, Tehran, Iran; ^4^School of Nutritional Sciences and Dietetics, Tehran University of Medical Sciences (TUMS), Tehran, Iran; ^5^School of Iranian Traditional Medicine, Tehran University of Medical Sciences, Tehran, Iran

##### **Correspondence:** Soodeh Razeghi Jahromi(soodehrazeghi@gmail.com); Mansoureh togha(Email: togha1961@gmail.com)

**Abstract:**

**Introduction:** Diet is recognized as a possible potential factor in migraine pathogenesis. As fruits and vegetables are considered as major sources of vitamins, minerals and dietary antioxidant, numerous studies have suggested them effective in various types of chronic disease. Limited evidence exists on the effect of diet on pediatric migraine, so we aimed to investigate the association between fruit and vegetable consumption and odds of migraine in children

**Method:** We conducted a case-control study in tertiary hospital, Tehran, Iran. One hundred children with migraine as case group and 190 sex-matched healthy controls were recruited in this study. Definite diagnosis of migraine was based on 2018 international classification of headache disorder 3 (ICHD3) criteria and confirmed by a neurologist. Demographic and anthropometric characteristics were collected. Common dietary intake of participants was obtained using a validated semi-quantitative food frequency questionnaire (FFQ). In order to run logistic regression models, all variables were classified into tertiles except for dietary fiber which was stratified into quartiles.

**Result:** Children in the migraine group had significantly higher BMI and age compared with the control group (P-value=0.00). After adjustment for age, gender, BMI and total energy intake a significant association between higher intake of vegetables in second tertile (OR: 0.47;CI:0.24-0.92), fruits in third tertile (OR: 0.31;CI:0.14-0.69) and fiber in fourth quartile (OR: 0.28;CI:0.095-0.85) was obtained. By controlling for all confounders in model 3, the odds of migraine, decreased by 50% and 70% by increasing in vegetables and fruits consumption, in the second tertile of vegetables (P-value=0.04) and the third tertile of fruits (P-value=0.00). We noticed 50% and 70% reduction in odds of assigning to migraine group by increasing the consumption of vegetable and fruit to 218/5 gr/week and 578/85 gr/week in the second tertile of vegetable and third teritle of fruit compared with the first tertile, respectively. Also a 72% reduction in odds of migraine was observed by an incremented level of fiber intake to 46/43 gr /week in the fourth quartile of dietary fiber.

**Conclusion:** Our findings confirm a plausible protective role of dietary fruits and vegetables against the risk of migraine in children which can be attributed to the probable effect of vitamins, minerals and also dietary fiber.

**Keywords:** Migraine, Pediatrics, fruits, vegetables, odds

## A50 Excessive daytime sleepiness in tension-type headache: a population-based study

### Tae-Jin Song^1^, Kyung Min Kim^2^, Dong Hyun Lee^2^, Won-Joo Kim^3^, Soo-Jin Cho^4^, Kwang Ik Yang^5^, Chang-Ho Yun^6^, Min Kyung Chu^2^

#### ^1^Department of Neurology, Severance Hospital, Yonsei University College of Medicine, Seoul, Korea; ^2^Department of Neurology, Severance Hospital, Yonsei University College of Medicine, Seoul, Korea; ^3^Department of Neurology, Gangnam Severance Hospital, Yonsei University College of Medicine, Seoul, Korea; ^4^Department of Neurology, Dongtan Sacred Heart Hospital, Hallym University College of Medicine, Hwaseong, Korea; ^5^Department of Neurology, Soonchunhyang University College of Medicine, Cheonan Hospital, Cheonan, Korea; ^6^Department of Neurology, Bundang Clinical Neuroscience Institute, Seoul National University Bundang Hospital, Seongnam, Korea

##### **Correspondence:** Tae-Jin Song(knstar@ewha.ac.kr)

**Introduction**

Excessive daytime sleepiness (EDS) is defined as ‘sleepiness in a situation when an individual would be expected to be awake and alert’ and has been reported to be associated with several neurological disorders including headache disorders. Tension-type headache (TTH) is the most common headache disorder in general population. Owing to its higher prevalence, the social burden of TTH is greater than that caused by migraine. Previous study has demonstrated that EDS was prevalent among migraineurs and was associated with an exacerbation of migraine. Nevertheless, information on the association between EDS and TTH is limited. The aim of this study is to investigate the association and impact of EDS on participants with TTH in a population-based setting.

**Methods**

This study used the data of the Korean Headache-Sleep Study (KHSS), which was a population-based survey regarding headache and sleep for Korean adults aged 19–69 years. If the score on the Epworth Sleepiness Scale (ESS) was more than or equal to 11, the participant was classified as having EDS.

**Results**

Of the 2695 participants, 570 (21.2%) and 313 (11.6%) were classified as having TTH and EDS, respectively. EDS was more prevalent among TTH participants with ≥15 headache frequency per month compared with participants with non-headache (35.7% vs. 9.4%, p < 0.001). Prevalence of EDS among TTH participants with headache frequency <1 per month (8.3%, p = 0.511) and TTH participants with headache frequency 1–14 per month (13.5%, p = 0.054) was not significantly different from that of those with non-headache. TTH participants with EDS had higher headache frequency per month (4.3 ± 8.1 vs. 1.7 ± 4.2, p = 0.013), Visual Analogue Scale for headache intensity (5.0 [3.0 – 6.0] vs. 4.0 [3.0 – 6.0], p = 0.008), Headache Impact Test-6 score (47.1 ± 7.3 vs. 43.5 ± 7.6, p < 0.001) and more depression (Patient Health questionnaire score≥10) (12.7% vs. 3.2%, p < 0.001) compared to those without EDS. Multivariable logistic regression revealed that headache frequency (β=0.051, p=0.016), Headache Impact Test-6 score (β=0.051, p=0.016), and depression (β=1.230, p=0.011) were independently associated with the existence of EDS among TTH participants.

**Conclusions**EDS is more prevalent in TTH participants with >15 headache frequency per month compared to those with non-headache. TTH participants with EDS was independently associated with higher headache frequency, increased impact of headache and higher prevalence of depression compared to those without EDS.

## A51 Hemiplegic migraine with late-onset cerebellar signs as phenotypic variant of CANCA1A mutation. Case Report

### Giagkou Eirini, Breza Marianthi, Natsis Vasileios, Mavridis Theodoros, Tountopoulou Argyro, Vassilopoulou Sofia, Mitsikostas Dimos Dimitrios

#### 1st Neurology Department, Eginition Hospital, School of Medicine, National and Kapodistrian University of Athens, Athens, Greece

##### **Correspondence:** Mavridis Theodoros(mavridismdr@gmail.com)

**Βackground**

Hemiplegic migraine (HM) is a rare and complex clinical entity which is characterized by aura consisting of fully reversible motor weakness as well as visual, sensory and/or speech/language symptoms and is categorized as familial (FHM) when at least one first- or second-degree relative experiences attacks of HM. In some cases, genes that prove causality have been identified (CACNA1A, ATP1A2, SCN1A). Although patients with mutations in CACNA1A may present with cerebellar ataxia, the presence of both clinical entities and moreover the emergence of late-onset cerebellar symptoms in a patient with known FHM, is extremely rare.

**Case report**

We present a case of 32-year-old man with a long-standing diagnosis of sporadic HM treated successfully with valproic acid who reports a mild difficulty in walking and a slight change in his speech, starting from 2 years ago. Neurological examination revealed cerebellar signs of mild dysarthria/scanning speech, mild gait ataxia (difficulty in tandem walking test), and dysdiadochokinesia while neuroimaging studies revealed cerebellar atrophy. The rest laboratory testing was without any pathological finding.

**Results**

The following genetic tests for mutations in spinocerebellar ataxia related genes (SCA panel) were all negative. Finally, a genetic study of FHM was performed and a heterozygous point mutation in CACNA1 gene (c.4046G>A, pArg1349Gln) was revealed, supporting the above findings.

**Discussion**

Whereas changes in ion channels may provide an explanation for aura symptoms (cortical hyperexcitability) and cerebellar degeneration (Purkinje cell expression), the pathophysiology and clinical presentation of CANCA1A mutations remain unknown. It is proposed that in any newly diagnosed case of HM, especially when cerebellar signs are present, appropriate genetic testing should be performed even in the absence of positive family history.

Written, informed consent for publication was obtained from the patient.

## A52 Multi-sensory gain abnormalities in postural tachycardia syndrome exceed those of migraine

### Melissa M. Cortez, Leah Millsap, K.C. Brennan

#### Department of Neurology, University of Utah

##### **Correspondence:** K.C. Brennan(k.c.brennan@hsc.utah.edu)

**Background:** Up to 90% of postural tachycardia syndrome (PoTS) patients report frequent, problematic headaches. According to current diagnostic criteria for PoTS, associated headaches might be expected to be strictly orthostatic, though comorbid migraine headache is common. Given this observation, it is possible that the two disorders interact pathophysiologically.

**Methods:** 30 PoTS, 30 chronic migraine (CM), and 30 non-headache (NH) control subjects completed a migraine questionnaire, MIDAS/HIT6, and sensory/autonomic symptom assessment. Sensory testing included mechanical pain thresholds by von Frey Hair and quantitative light sensitivity thresholds.

**Results:** Photophobia and allodynia symptom scores were significantly higher in both CM and PoTS groups compared to controls. COMPASS-31 autonomic symptom scores were significantly higher in PoTS compared to CM, but both groups had significantly higher scores than NH. Unexpectedly, photophobia thresholds were significantly lower in PoTS compared to CM; both PoTS and CM thresholds were significantly decreased compared to NH. Mechanical pain thresholds were also significantly lower in both CM and PoTS compared to NH. Here the patterns of expression differed between CM and PoTS, with reduced periorbital and forearm pain thresholds in CM, and only forearm threshold reductions in PoTS subjects.

**Discussion:** We found evidence of ‘migraine-like’ sensitization and autonomic symptoms in both CM and PoTS subjects, suggesting common pathway activation in these often-comorbid conditions. However, we also observed distinguishable features in each disorder that likely reflect divergent network responses. We found that PoTS subjects (including PoTS subjects without migraine) had significantly lower light sensitivity thresholds, and a differing pattern of allodynia, compared to CM. This reveals an under-appreciated aspect of disease burden in PoTS, and suggests network sensitization similar to, but separable from that of migraine.

## A53 Healthcare resource utilization in adult patients treated with onabotulinumtoxinA for chronic migraine: results from the COMPEL study

### John F. Rothrock^1^, Richard Stark^2^, Katherine Sommer^3^, Andrew M. Blumenfeld^4^

#### ^1^George Washington University School of Medicine, Washington, DC, USA, ^2^Alfred Hospital and Monash University, Melbourne, Australia, ^3^Allergan plc, Marlow, UK, ^4^Headache Center of Southern California, The Neurology Center, Carlsbad, CA, USA

##### **Correspondence:** Katherine Sommer(sommer_kathrine@allergan.com)

**Background:** This subanalysis of the COMPEL study evaluates health resource utilization (HRU) by patients treated with onabotulinumtoxinA for chronic migraine (CM).

**Methods:** The single-arm, open-label, multicenter, prospective study (NCT01516892) enrolled adults with CM receiving onabotulinumtoxinA 155 U approximately every 12 weeks (9 treatments, 108 weeks). Patients recorded headache (HA) days in a daily diary used to assess change from baseline (BL) in HA days/28 days. HRU data (total HA-related visits to a healthcare professional [HCP], emergency room [ER]/urgent care [UC] visits, overnight hospital stays, and number of HA-related diagnostic tests) were collected at BL (for past 6 months) and each treatment session (since last visit).

**Results:** Mean age of patients (N=716) was 43 years; 85% were female. At BL, 557 patients reported a mean (SD; range) of 7.0 (15.4; 0-198) HA-related visits to an HCP. Statistically significant reductions in mean (SD) visit frequency occurred at all visits: 3.9 (7.5), week 24; 3.4 (5.4), week 48; 2.9 (4.2), week 72; and 3.3 (6.8), week 96. At each visit, a majority of patients reported seeing a neurologist/HA specialist. At BL, 690 patients reported a mean (SD) of 0.5 (2.0) HA-related ER/UC visits; 691 reported 0.2 (1.5) HA-related overnight hospital stays. After onabotulinumtoxinA treatment, HA-related ER/UC visits decreased at each visit. Statistically significant reductions in HA-related ER/UC visits and overnight hospital stays were reported at all visits. At BL, 193 patients reported having had 487 HA-related diagnostic tests; 334 tests were reported from weeks 24 to 96.

**Discussion:** Real-world data from the COMPEL study show that onabotulinumtoxinA therapy is associated with reductions in HRU, including HA-related visits to an HCP, ER/UC visits, overnight hospital stays, and total number of diagnostic tests, supporting long-term benefits associated with use of onabotulinumtoxinA for treating CM in clinical practice.

**Disclosure of Support:** Allergan plc, Dublin, Ireland

## A54 Effects of prolonged treatment with single pulse Transcranial Magnetic Stimulation (sTMS) in animal models of migraine

### M Jones^2,3,^ S McMahon^2^, R Abuukar Abdullahi^1,4^, G Lambru^4^, AP Andreou^1,4^

#### ^1^Headache Research-Wolfson CARD, ^2^Neurorestoration Department-Wolfson CARD, King's College London, ^3^Zenith Neurotech Ltd, 4Headache Centre, Guy's and St Thomas' NHS Trust, London, United Kingdom

##### **Correspondence:** AP Andreou(*joseph.lloyd@kcl.ac.uk*)

**Background:** Single-pulse transcranial magnetic stimulation (sTMS) is a non-invasive neuromodulation technique shown to be successful for migraine treatment. Previous research has shown that acute sTMS treatment increases the threshold of cortical spreading depression (CSD) induction, but it does not influence trigeminal nociception. Its long-term effects have not been previously investigated.

**Methods:** Daily treatments of either 1.1 T or 0 T sTMS were applied to awake male adult Sprague- Dawley rats for a period of 30 days using a custom made sTMS coil with a diameter of 11 mm and stimulating parameters similar to the migraine treatment. Behavioural testing of paraorbital von Frey thresholds was performed every other day for the first two weeks followed by every 5th day until day 30. At the conclusion of daily-treatments, CSD induction thresholds by electrical stimulation of the occipital cortex were determined in anaesthetised animals. All procedures were performed under a UK Home Office Licence in accordance to the 1986 Animal (Scientific Procedures) Act.

**Results:** Thirty days of daily treatment with 1.1 T sTMS induced no significant changes in the paraorbital von Frey threshold compared to the control group (0 T sTMS) (F1, 78 = 1.337, p<.251). CSD induction thresholds were found to be significantly increased in the 1.1 T sTMS treatment group compared to the 0 T controls (F1, 14 = 7.53, p<.016).

**Discussion:** Daily sTMS treatment did not alter mechanical von Frey thresholds suggesting it does not sensitize the trigeminal system. The increase in the CSD threshold in the active treatment group does suggest that sTMS has a long-term cumulative effect in inhibiting cortical excitation.

## A55 DEPRESSION AND ANXIETY LIKE SYMPTOMS IN PRIMARY HEADACHE DISORDERS

### Lypiridou M, Belesioti I, Deligianni C, Mitropoulou E, Kasioti E, Mitsikostas DD

#### ^1^First Neurology Department, Aeginition Hospital, Medical School, National & Kapodistrian University of Athens, Athens, Greece

##### **Correspondence:** Lypiridou M(marialypiridou@gmail.com )

**Background:** There is strong evidence that primary headache disorders (PHD), the chronic subtypes in particular, often comorbid with depression and other affective disorders (Mitsikostas and Thomas, 1999).

**Aim:** To investigate the frequency of depression and anxiety-like symptoms in patients with PHDs.

**Methods:** Patients attending the headache outpatient clinic were prospectively recruited to participate in the study. All underwent special interview with the Hamilton Depression Rating Scale (HAM-D) and Hamilton Anxiety Rating Scale (HAM-A). PHD diagnosis was performed according to the ICHD-3beta.

**Results:** 2070 consecutive headache outpatients were interviewed (1452 women, 70.1% and 618 men, 29.9%). 933 (45,1%) were suffering from chronic PHD and 1137 (54,9%) from episodic PHD subtypes; 1000 patients (48,3%) were suffering from migraine (720 (34,8%) episodic and 280 (13,5%) chronic), 761 (36,8%) from tension type headache (TTH) (341 (16,5%) episodic and 420 (20,3%) chronic) and 442 (21,4%) from medication overuse headache (MOH).

Patients suffering from chronic PHDs showed higher scores for both HAM-A (OR= 1.8) and HAM-D (OR= 1.54) than those suffering from episodic PHDs. The same for patients suffering from MOH vs. no MOH patients (OR=2.13 for anxiety, OR= 2.38 for depression). In migraine patients HAM-A and HAM-D scores were higher than those suffering from cluster headache (OR=3.01, OR=2.30). On the other hand migraine patients have 30% and 24.5% lower odds to score higher on the anxiety scale and depression scale respectively, compared to the TTH patients (OR=1.43, OR=1.32).

**Conclusion:** Outpatients suffering from chronic PHDs, TTH, migraine or MOH need further evaluation for potential mood comorbidities that require special care.

